# Soy protein alleviates DKD by restraining inflammation via the MAPKs/PPAR-γ signaling pathway

**DOI:** 10.1080/0886022X.2026.2698775

**Published:** 2026-07-24

**Authors:** Ying Zhang, Jing Xie, Minhui Wang

**Affiliations:** ^a^ Department of Nephrology, Hainan General Hospital (Hainan Affiliated Hospital of Hainan Medical College), Haikou, China; ^b^ Department of Pharmacy, Hainan General Hospital (Hainan Affiliated Hospital of Hainan Medical College), Haikou, China

**Keywords:** Diabetic Kidney Disease, soy protein, genistein, MAPKs/PPAR-γ signaling pathway, inflammation, oxidative stress

## Abstract

**Background:**

Diabetic Kidney Disease (DKD), a leading cause of kidney failure driven by chronic inflammation and dysregulated signaling, lacks effective therapies. This study explored if soy protein alleviates DKD *via* the MAPKs/PPAR-γ pathway.

**Methods:**

Bioinformatics on GEO datasets (GSE154881, GSE139317) identified differentially expressed genes (DEGs): GSE154881 (peripheral blood) had 580 DEGs enriched in inflammation/MAPK signaling; GSE139317 (kidney tissues) had 2830 DEGs enriched in fatty acid metabolism/PPAR signaling, suggesting MAPK-PPAR-γ crosstalk. Although soy isoflavones have been reported to modulate MAPK or PPAR-γ signaling in other diseases, whether soy protein affects this pathway crosstalk in DKD remains unknown. Based on the bioinformatic prediction, we hypothesized that soy protein ameliorates DKD by targeting the MAPKs/PPAR-γ axis.

**Results:**

In streptozotocin-induced DKD mice, soy protein (200–800mg/kg) dose-dependently reduced serum creatinine, blood urea nitrogen, blood glucose, tubular injury, kidney pro-inflammatory cytokines (MCP-1, IL-6, TNF-α), and reversed weight loss. In high glucose-stimulated HK-2 cells, genistein (soy isoflavone, 20–100μM) dose-dependently restored viability, reduced MDA (oxidative stress), increased GSH (antioxidant), and lowered cytokines. p38 MAPK agonist (diprovocim) or PPAR-γ antagonist (GW9662) abolished these effects.

**Conclusion:**

Soy protein alleviates DKD *via* genistein, which inhibits p38 MAPK and activates PPAR-γ to reduce inflammation/oxidative stress, supporting soy-based DKD interventions.

## 1. Introduction

Diabetic Kidney Disease (DKD) stands as a leading cause of kidney failure globally, imposing a substantial burden on public health systems and significantly impairing the quality of life of patients with type 2 diabetes (T2D) [1–3]. The pathogenesis of DKD is characterized by a complex interplay of metabolic, chronic inflammation, and kidney structural remodeling-including tubular injury, interstitial fibrosis, and glomerular sclerosis [2–6]. Among the key molecular cascades driving DKD progression, the mitogen-activated protein kinases (MAPKs) pathway plays a pivotal role in transducing inflammatory signals and mediating kidney cell damage, while peroxisome proliferator-activated receptor-γ (PPAR-γ) exerts protective effects by regulating lipid metabolism, suppressing inflammation, and maintaining kidney homeostasis [7,8]. Dysregulation of the MAPKs/PPAR-γ signaling axis has been increasingly recognized as a critical contributor to the exacerbation of DKD, making it a potential therapeutic target for intervention.

Despite advances in glucose control and antihypertensive therapies, the clinical management of DKD remains suboptimal, highlighting the need for novel, safe, and effective therapeutic strategies [9,10]. In recent years, soy-derived bioactive compounds-particularly soy protein and its major isoflavone component, genistein-have gained attention for their potential renoprotective properties [11–13]. Epidemiological studies have suggested an association between soy-rich diets and reduced risk of kidney dysfunction in people with diabetes, while preclinical investigations have demonstrated that soy protein can ameliorate hyperglycemia-induced kidney injury by modulating oxidative stress and inflammatory responses [14,15]. However, the precise molecular mechanisms underlying the beneficial effects of soy protein in DKD-especially its potential interaction with the MAPKs/PPAR-γ signaling pathway-remain incompletely elucidated.

We therefore hypothesized that soy protein and its key isoflavone genistein exert renoprotective effects in DKD by suppressing MAPK-mediated pro-inflammatory signaling and activating PPAR-γ, thereby reducing oxidative stress, tubular injury, interstitial fibrosis, and epithelial-mesenchymal transition (EMT). To test this hypothesis, a multi-faceted approach was employed in the present study. First, bioinformatics analyses of public gene expression datasets (GSE154881 and GSE139317) from the Gene Expression Omnibus (GEO) database were conducted to confirm the association between MAPK and PPAR-γ signaling pathways in DKD, thereby providing a theoretical basis for subsequent experimental validation. Subsequently, *in vivo* experiments using a streptozotocin (STZ)-induced DKD mouse model were performed to evaluate the effects of soy protein supplementation on kidney function and histopathological changes [16–18]. Complementary *in vitro* studies were conducted using high glucose (HG)-stimulated human proximal tubular epithelial cells (HK-2 cells)—a well-established model of renal tubular injury in DKD—to dissect the role of genistein in modulating oxidative stress and the secretion of pro-inflammatory cytokines, including monocyte chemoattractant protein-1 (MCP-1), interleukin-6 (IL-6), and tumor necrosis factor-alpha (TNF-α).

Crucially, to confirm the involvement of the MAPKs/PPAR-γ pathway, pharmacological interventions were utilized: Diprovocim (a p38 MAPK agonist) and GW9662 (a selective PPAR-γ antagonist) were employed to manipulate pathway activity in HG-stimulated HK-2 cells. By assessing changes in oxidative stress, inflammation, and EMT markers following these interventions, we aimed to validate whether soy protein/genistein exerts its reno-protective effects by targeting the MAPKs/PPAR-γ ­signaling axis.

Collectively, this study sought to: (1) identify key molecular pathways dysregulated in DKD *via* bioinformatics analysis; (2) verify the reno-protective effects of soy protein in a DKD mouse model; (3) elucidate the role of genistein in mitigating HG-induced renal tubular cell injury; and (4) confirm the regulatory role of the MAPKs/PPAR-γ pathway in mediating the beneficial effects of soy-derived compounds. The findings of this study may provide novel insights into the molecular mechanisms of DKD and support the development of soy-based therapeutic strategies for the prevention and treatment of this devastating disease.

## 2. Materials and methods

### 2.1. Bioinformation analysis

We retrieved microarray datasets GSE154881 and GSE139317 from the GEO database to explore gene expression alterations in DKD. The GSE154881 dataset, based on platform GPL24676, comprised peripheral blood samples from healthy controls, patients with T2D, and individuals diagnosed with DKD. This study was approved by the Ethics Committee of Shanghai Jiao Tong University Affiliated Sixth People’s Hospital, all participants provided written informed consent before sample collection, and all procedures adhered to the principles of the Declaration of Helsinki.

The GSE139317 dataset, profiled on platform GP21163, included kidney tissues from mice treated with either Vehicle (Sham group; *n* = 6) or 90 mg/kg alloxan administered intravenously three days prior to grouping. All bioinformatic analyses were performed using R Studio. Differential gene expression analysis was carried out with the limma package, with differentially expressed genes (DEGs) defined using an adjusted P value < 0.05. Functional enrichment analyses, including Gene Ontology (GO) and Kyoto Encyclopedia of Genes and Genomes (KEGG) pathways, were conducted using the ‘enrichplot’ and ‘ggplot2’ packages in R, with a significance threshold of *p* < 0.05.

The Search Tool for the Retrieval of Interacting Genes (STRING; http://string-db.org/) database specializes in evaluating protein-protein interaction (PPI) information. In this study, the STRING online platform was employed to construct PPI networks for DEGs, with a combined interaction score > 0.4 set as the threshold for statistically significant interactions. Among the available algorithms, we selected the Maximal Clique Centrality (MCC) method for identifying essential hub nodes in biological networks, as it considers both the number and the interconnectivity of a node’s neighbors [19]. Based on the MCC scores, the top 10 ranked genes were selected as the key targets for further investigation.

### 2.2. Animal studies

C57BL/6 mice (6–8 weeks old) were maintained under specific pathogen-free conditions (three mice per cage) at 22 ± 2 °C and 50 ± 5% relative humidity with a 12-h light-dark cycle, with free access to food and water throughout the study. The animals were obtained from the Animal Center of Hainan Medical College University. No previous procedures, treatments, or surgeries had been performed on these animals prior to the start of this experiment.

Based on previous studies in similar animal models, a minimum of 6 animals per group was considered sufficient to detect a biologically relevant difference with acceptable variability [20,21]. Randomization was performed using a computer-generated random number sequence (R Studio, sample function with a fixed seed of 12345). Specifically, each mouse was assigned a unique number. The R function generated a random permutation of these numbers; the first six numbers were allocated to the control group, and the remaining numbers were sequentially allocated to the four DKD model groups. The mice were randomly divided into five groups (*n* = 6 per group). The control group received sodium citrate buffer and a standard chow diet. The experimental group was fed a high-fat diet (HFD) for 6 weeks followed by intraperitoneal administration of STZ at 40 mg/kg for five consecutive days to induce DKD as previously described. Three DKD model groups then received soy protein *via* intragastric gavage (i.g.) at doses of 200 mg/kg daily, 400 mg/kg every three days, or 800 mg/kg every three days, respectively. After 8 weeks of intervention, successful DKD modeling was confirmed by measuring blood glucose levels exceeding 16.7 mmol/L [22], four mice that did not meet this criterion were excluded. All animals were euthanized *via* cervical dislocation under anesthesia induced by intraperitoneal injection of sodium pentobarbital.

The study was conducted in a blinded manner. Group allocation was concealed from the researchers performing the interventions throughout the 8-week treatment period. Furthermore, the investigators responsible for the subsequent biochemical and histopathological evaluations remained blinded to group assignment until the final data analysis was completed. All procedures conformed to the institutional guidelines for animal care and were approved by the Medical Ethics Committee of Hainan Provincial People’s Hospital (Approval No. EC-YLY-2025-206-01).

### 2.3. Cell culture and treatment

The HK-2 cells line was obtained from the China Cell Bank and maintained in DMEM/F12 medium (Gibco, USA, Cat. No. 11966025) supplemented with 10% fetal bovine serum (FBS; Gibco, USA, Cat. No. 10099-141) and 1% penicillin-streptomycin (Thermo Fisher Scientific, Canada, Cat. No. 15070063). Cells were cultured at 37 °C in a humidified atmosphere containing 5% CO_2_.

The experimental groups consisted of the following treatments: 5 mM glucose (Control), 30 mM mannitol (Man), and 30 mM D-glucose (HG). Genistein was prediluted to final concentrations of 20, 50, and 100 μM and administered to the cells 30 min prior to HG exposure. To investigate the involvement of the MAPK/PPAR-γ pathway, HK-2 cells under HG conditions were pretreated for 2 h with 0.5 μM Diprovocim (HY-123942, MedChemExpress, (MCE), China), a p38 MAPK agonist. In addition, 10 μM of GW9662 (HY-16578, MCE), a potent antagonist of PPARγ, was treated to HG-treated HK-2 cells and incubated for 48 h.

### 2.4. Biochemical analyses

Serum levels of creatinine (Scr), urea nitrogen (BUN), and glucose were quantified using an iChem-340 Autoanalyzer (iCubio, Shenzhen, China). The measurements were conducted employing commercial assay kits (BSBE, Beijing, China) based on the sarcosine oxidase method (GCRE460BSM) for Scr, the urea/glutamate dehydrogenase method (GUREA460BS) for BUN, and the glucose oxidase method (GLU460BS) for glucose, respectively.

### 2.5. Histopathology

Fresh mouse kidney tissues were fixed in 4% paraformaldehyde, embedded in paraffin, and sectioned at 4 μm thickness. Following dewaxing in xylene and rehydration through a graded ethanol series, the sections were subjected to hematoxylin and eosin (H&E) staining for evaluation of kidney histopathological changes. Periodic acid–Schiff (PAS) staining was additionally employed to examine glycogen deposition.

### 2.6. Determination of GSH and MDA levels

HK-2 cells were homogenized using an ultrasonic disruptor. GSH levels in cell lysates were assessed using a commercial glutathione assay kit (BC1175, Solarbio, China) following the manufacturer’s instructions, and the resulting concentrations were calculated based on a standard curve and normalized to total protein content. MDA levels were determined with a corresponding kit (S0131S, Beyotime, China) by measuring the absorbance at 535 nm.

### 2.7. Determination of MCP-1, IL-6, TNF-α levels

The concentrations of MCP-1 (E-EL-M3001; Elabscience, China), IL-6 (E-EL-M0044; Elabscience, China), and TNF-α (E-EL-M3063; Elabscience, China) in HK-2 cells culture supernatants were quantified using enzyme-linked immunosorbent assay according to the manufacturer’s protocols.

### 2.8. Quantitative real-time polymerase chain reaction (qRT-PCR)

Total RNA was extracted from renal cortex using an RNA isolation kit (Qiagen, Düsseldorf, Germany) according to the manufacturer’s instructions. Reverse transcription was performed to synthesize complementary DNA (cDNA) using the First Strand cDNA Synthesis Kit (K1642, Thermo Fisher Scientific, Rockford, IL, USA). Quantitative real-time PCR (qPCR) was carried out with Power SYBR^™^ Green Master Mix (4367659, Applied Biosystems, Carlsbad, CA, USA). All experiments were conducted in triplicate, and relative gene expression levels were calculated using the 2^−ΔΔCt^ method. The primer sequences for each gene are listed:

**Table:** 

Mouse Gene	Sequence (5′->3′)
F- p38 MAPK	TGACCCTTATGACCAGTCCTTT
R- p38 MAPK	GTCAGGCTCTTCCACTCATCTAT
F- PPAR-γ	GGAAGACCACTCGCATTCCTT
R- PPAR-γ	GTAATCAGCAACCATTGGGTCA

### 2.9. Western blot analysis

HK-2 cells were lysed in RIPA buffer (C1053, Applygen, Beijing, China) containing a protease inhibitor cocktail and phenylmethanesulfonyl fluoride (PMSF). Protein concentration was determined using a BCA protein assay kit (P0010, Beyotime Biotechnology, China). The samples were separated by sodium dodecyl sulfate-polyacrylamide gel electrophoresis (SDS-PAGE) and transferred onto polyvinylidene difluoride (PVDF) membranes (GE Healthcare, Buckinghamshire, UK). The membranes were blocked with 10% nonfat milk and incubated overnight at 4 °C with the following primary antibodies: α-SMA (1:1000; #14968, Cell Signaling Technology (CST), USA), Collagen Type I (1:1000; 67288-1-Ig, proteintech, China). Subsequently, the membranes were incubated with horseradish peroxidase (HRP)-conjugated goat anti-rabbit IgG secondary antibody (1:4000, 31460, Thermo Fisher Scientific, Marietta, GA, USA) for 1 h at RT. ImageJ software (NIH, USA) was used for analyzing the bands.

### 2.10. Statistical analysis

All cell experiments were performed at least three times, while all animal experiments were performed at least six times. GraphPad Prism software version 9.0 was used for statistical analysis. Quantitative data are expressed as the mean ± standard error of the mean (SEM), following confirmation of normality and homogeneity of variance. Statistical analyses were performed using T-test or one-way analysis of variance, followed by all pairwise multiple comparison procedures using the Bonferroni test. Statistical significance was set at *p* < 0.05.

## 3. Result

### 3.1. Comparative analysis of blood-derived transcriptomes reveals DGEs in T2D and DKD

**Figure 1.**
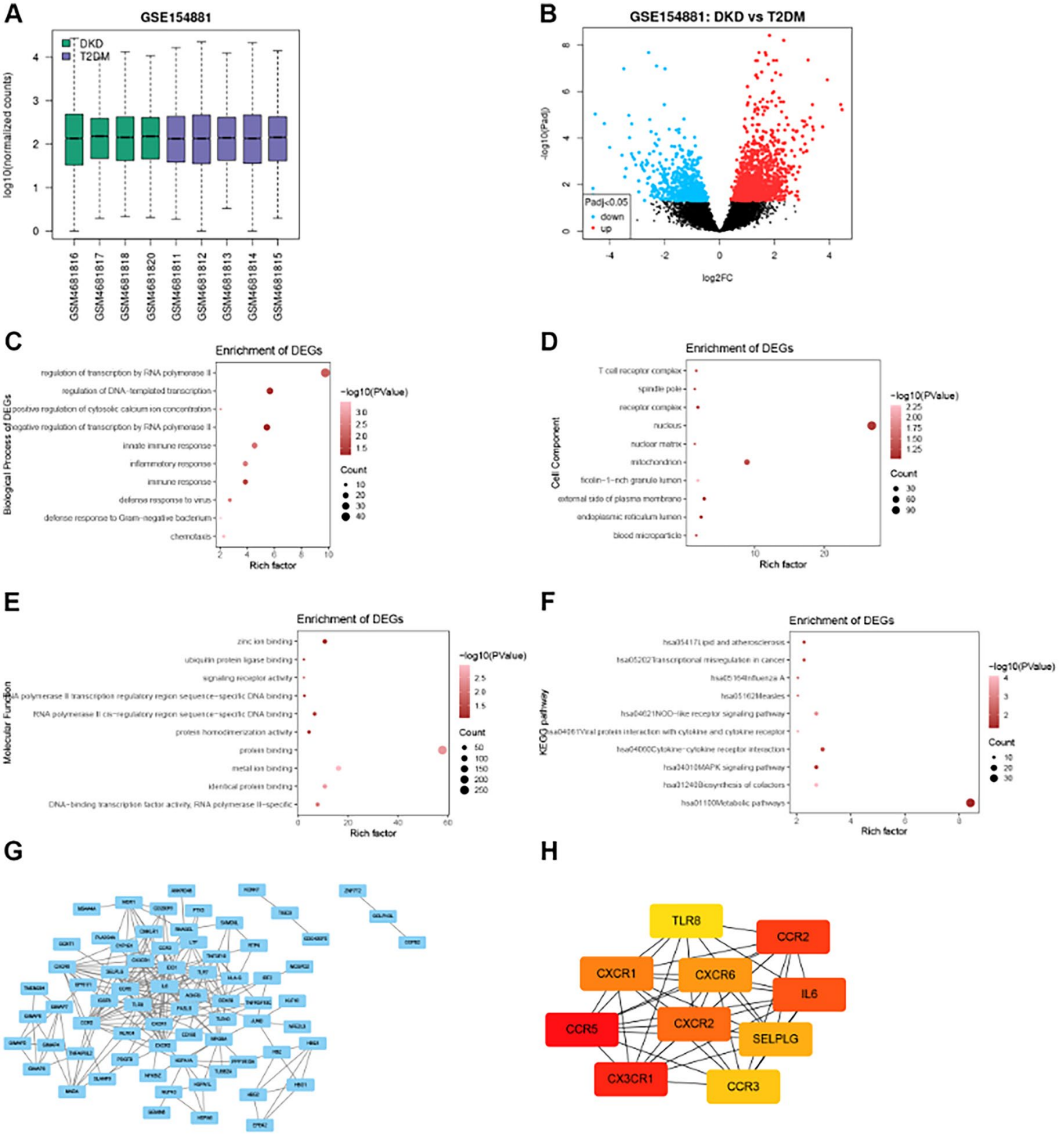
Identification of DEGs and functional enrichment analysis in GSE154881. (A) A boxplot of GSE154881. (B) A volcano plot of DEGs in GSE154881. (C-F) GO analysis and KEGG pathway enrichment analysis of DEGs in GSE154881. (G) The PPI between DEGs is analyzed using Cytoscape in GSE154881. (H) The top 10 key genes were screened through the PPI network map in GSE154881.

In the GSE154881 dataset, peripheral blood samples from healthy controls, T2D, and DKD were analyzed. Differential gene expression analysis between T2D and DKD groups was conducted using the limma package in R, with normalized distribution visualized in a boxplot (Figure 1A). This comparison identified 395 upregulated and 185 downregulated DEGs (Figure 1B).

Functional enrichment analysis of these DEGs was performed through GO and KEGG annotations. GO analysis revealed significant enrichment across 53 biological processes (BPs), 7 cellular components (CCs), and 19 molecular functions (MFs). Key terms included chemotaxis, defense response to Gram-negative bacterium, regulation of interleukin production, and chaperone cofactor-dependent protein refolding in BPs; ficolin-1-rich granule lumen, protein phosphatase complex, blood microparticle, T cell receptor complex, and mitochondrion in CCs; as well as metal ion binding, pore-forming activity, protein binding, and ATP-dependent disaggregase activity in MFs (Figure 1C–E, Table S1). KEGG pathway analysis indicated that DEGs were predominantly associated with cofactor biosynthesis, viral protein–cytokine interactions, NOD-like receptor signaling, cytokine–cytokine receptor interaction, and MAPK signaling pathways (Figure 1F and Table S1).

To further explore functional interactions among DEGs, a PPI network was constructed using the STRING database and visualized in Cytoscape (Figure 1G). Top hub genes-including CCR5, CX3CR1, CCR2, IL6, CXCR2, CXCR1, CXCR6, SELPLG, CCR3, and TLR8 were identified employing the MCC algorithm *via* CytoHubba in Cytoscape (Figure 1H).

### 3.2. Soy protein alleviates tubular injury in DKD

**Figure 2.**
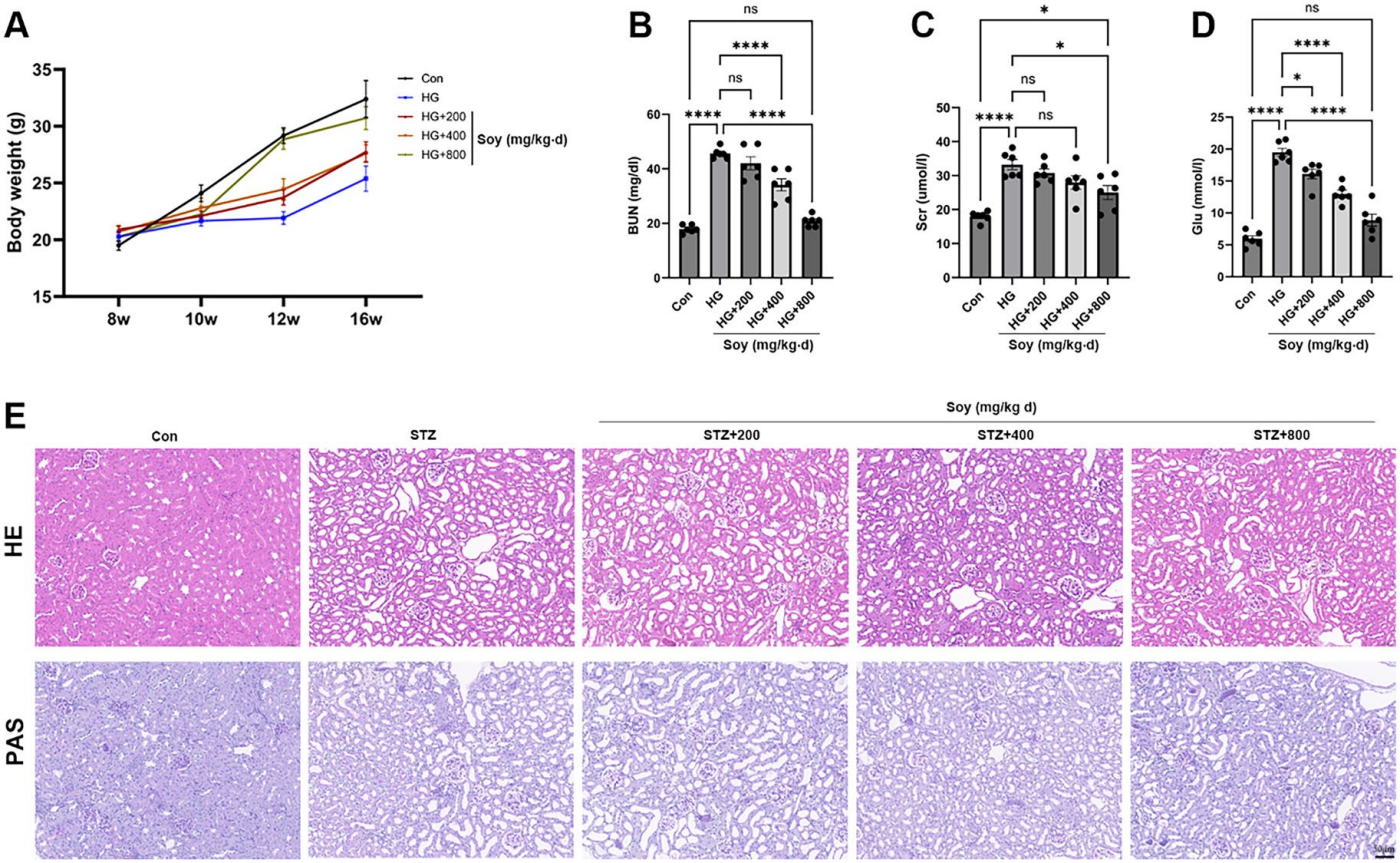
Soy protein alleviates tubular injury in DKD. (A–D) Body weight, BUN, Scr, and Glu levels in each group were determined, *n* = 6. (E) Mouse kidney sections were stained with HE and PAS, Scale bar = 50 μm. Data are presented as mean ± SEM. Asterisks indicate significant differences: * *p* < 0.05.

To elucidate the role of soy protein in DKD, key biochemical kidney parameters and histopathological features were assessed in STZ-induced diabetic mice. Body weight was substantially reduced in DKD, an effect that was reversed by soy protein diet, which occurred in a dose-times-dependent manner (*n* = 6, Figure 2A). Conversely, compared to the control group, BUN, SCr, and blood glucose levels were significantly elevated in DKD mice. However, DKD mice fed a soy protein diet exhibited marked improvements in the aforementioned parameters, which occurred in a dose-dependent manner (*n* = 6, Figure 2B–D).

Histopathological examination further revealed severe kidney damage in DKD mice, characterized by tubular epithelial cell shedding, loss of brush borders, pronounced tubular vacuolization, tubular swelling, and interstitial fibrosis. In contrast, soy protein diet treatment markedly ameliorated these pathological alterations in the DKD mice (Figure 2E and Figures S1, S2). Taken together, these results indicate that soy protein diet alleviates tubular injury in DKD mice.

### 3.3. Identification of DEGs and functional enrichment analysis in DKD mice

**Figure 3.**
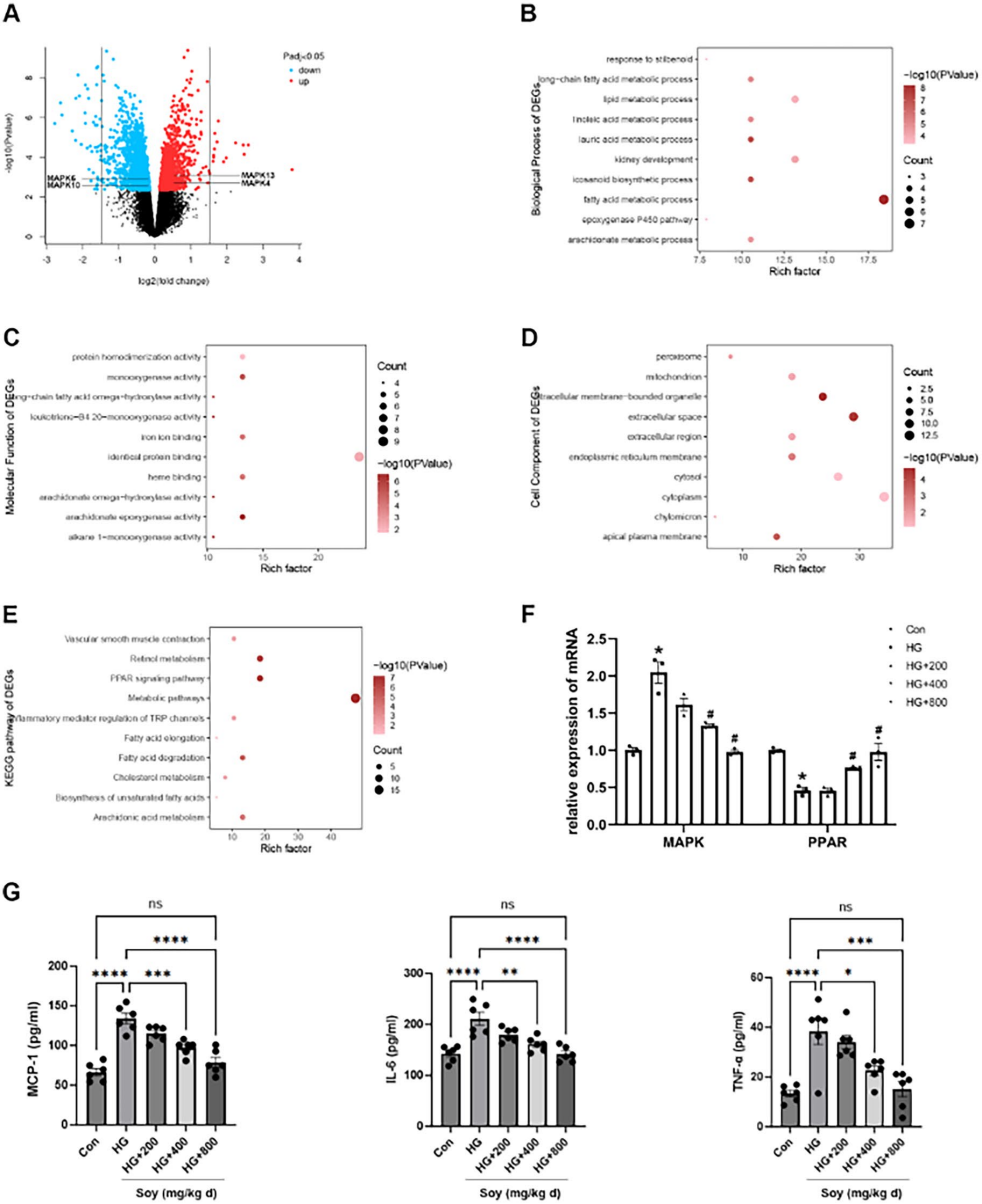
Identification of DEGs and functional enrichment analysis in GSE139317. (A) A volcano plot of DEGs in GSE139317. (B–E) GO analysis and KEGG pathway enrichment analysis of DEGs in GSE139317. (F) The mRNA expression of MAPK and PPAR were measured in kidney tissue of mice in each group. *n* = 3. (G) MCP-1, IL-6, and TNF-α levels in kidney tissue of mice in each group, *n* = 6. Data are presented as mean ± SEM. Asterisks indicate significant differences: * *p* < 0.05.

In GSE139317 dataset, kidney samples were collected and total RNAs from kidney tissues were analyzed on affymetrix microarrays. Sham and DKD group were selected for DEGs. In total, 1132 upregulated and 1698 downregulated DEGs were identified between DKD and sham group (Figure 3A). GO analysis revealed significant enrichment of DEGs in 24 BPs, 9 CCs, and 21 MFs, with the top five significant terms comprising: fatty acid metabolic process, lauric acid metabolic process, icosanoid biosynthetic process, long-chain fatty acid metabolic process, linoleic acid metabolic process (BPs); intracellular membrane-bounded organelle, extracellular space, apical plasma membrane, endoplasmic reticulum membrane, peroxisome (CCs); arachidonate epoxygenase activity, arachidonate omega-hydroxylase activity, alkane 1-monooxygenase activity, leukotriene-B4 20-monooxygenase activity, long-chain fatty acid omega-hydroxylase activity (MFs) (Figure 3B–D, and Table S2). KEGG analysis demonstrated that upregulated DEGs were primarily associated with metabolic pathways, PPAR signaling pathway, retinol metabolism, fatty acid degradation, arachidonic acid metabolism (Figure 3E and Table S2). Compared with the control group, DKD mice showed significantly elevated MAPK mRNA expression. In contrast, DKD mice fed a soy protein diet exhibited marked reductions in MAPK levels, whereas PPAR expression showed the opposite trend (*n* = 3, Figure 3F). Additionally, levels of MCP-1, IL-6, and TNF-α were significantly increased in DKD mice; however, administration of a soy protein diet led to substantial, dose-dependent improvements in these parameters (*n* = 6, Figure 3G).

### 3.4. Genistein attenuates the release of pro-inflammatory factors in HG-induced HK-2 cells

**Figure 4.**
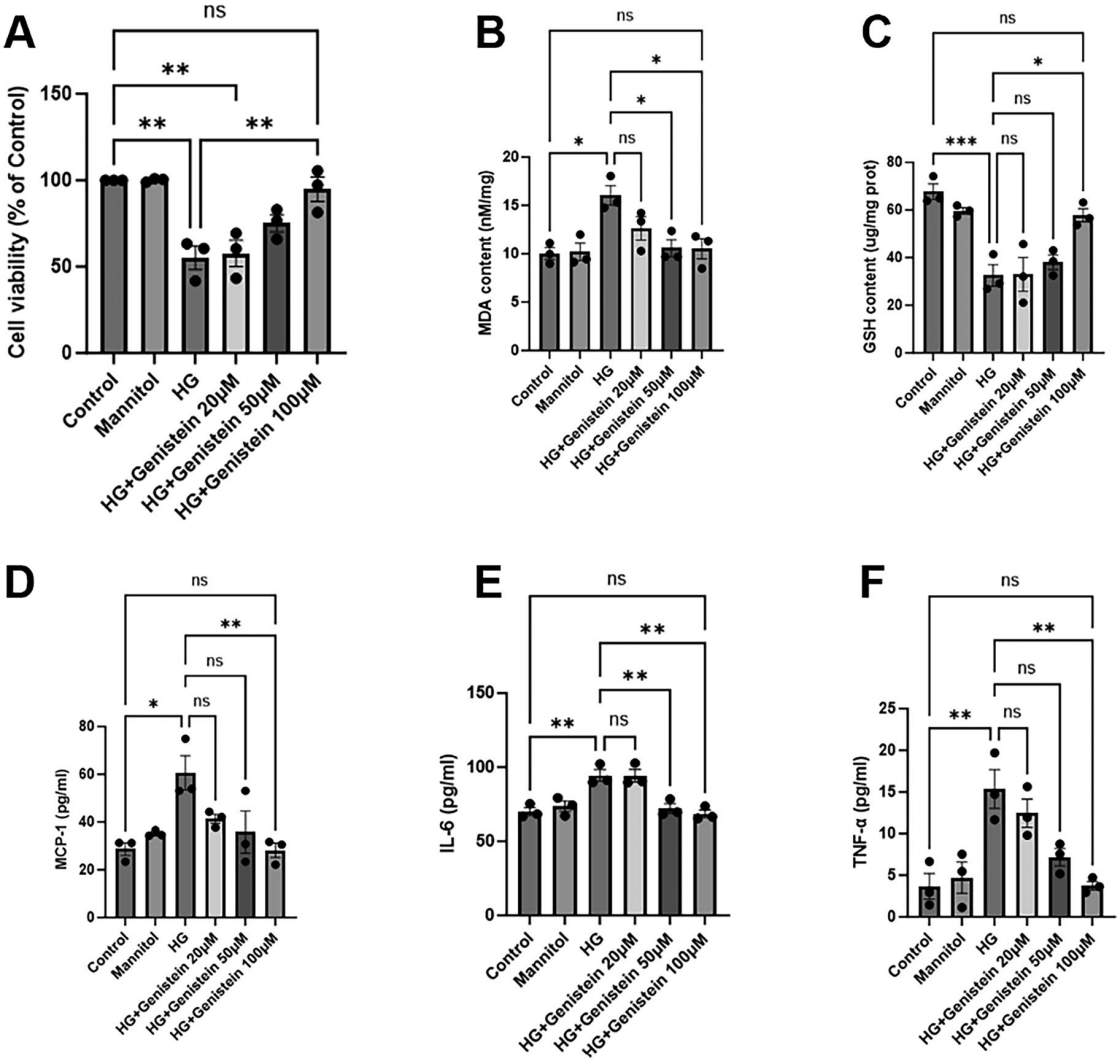
Genistein attenuates the release of pro-inflammatory factors in HG-induced HK-2 cells. (A–C) The cell viability, concentrations of MDA and GSH were detected in each groups, *n* = 3. (D–F) The MCP-1, IL-6, TNF-α were detected in each groups, *n* = 3. Data are presented as mean ± SEM. Asterisks indicate significant differences: * *p* < 0.05.

Genistein is an isoflavone first isolated from the brooming plant Dyer’s *Genista tinctoria L*, and is widely distributed in the *Fabaceae* family [23]. HK-2 cells were treated with varying concentrations of genistein under HG conditions. The results showed that HG exposure reduced cell viability, which was significantly restored by treatment with 20, 50, and 100 μM genistein for 48 h (*n* = 3, Figure 4A). Compared to the control group, MDA levels were markedly elevated in the HG group; however, this increase was attenuated following genistein treatment (*n* = 3, Figure 4B). Conversely, GSH levels were substantially decreased under HG conditions, and this reduction was reversed by genistein in a dose-dependent manner (*n* = 3, Figure 4C). Levels of MCP-1, IL-6, and TNF-α were significantly elevated in the HG group but were markedly reduced in the genistein-treated groups, also in a dose-dependent manner (*n* = 3, Figure 4D–F). These results suggest that the nephroprotective effect of soy-derived compounds may be mediated through the reduction of pro-inflammatory factor secretion.

### 3.5. Targeting the MAPK/PPAR-γ pathway mediates HG-stimulated EMT in HK-2 cells

**Figure 5.**
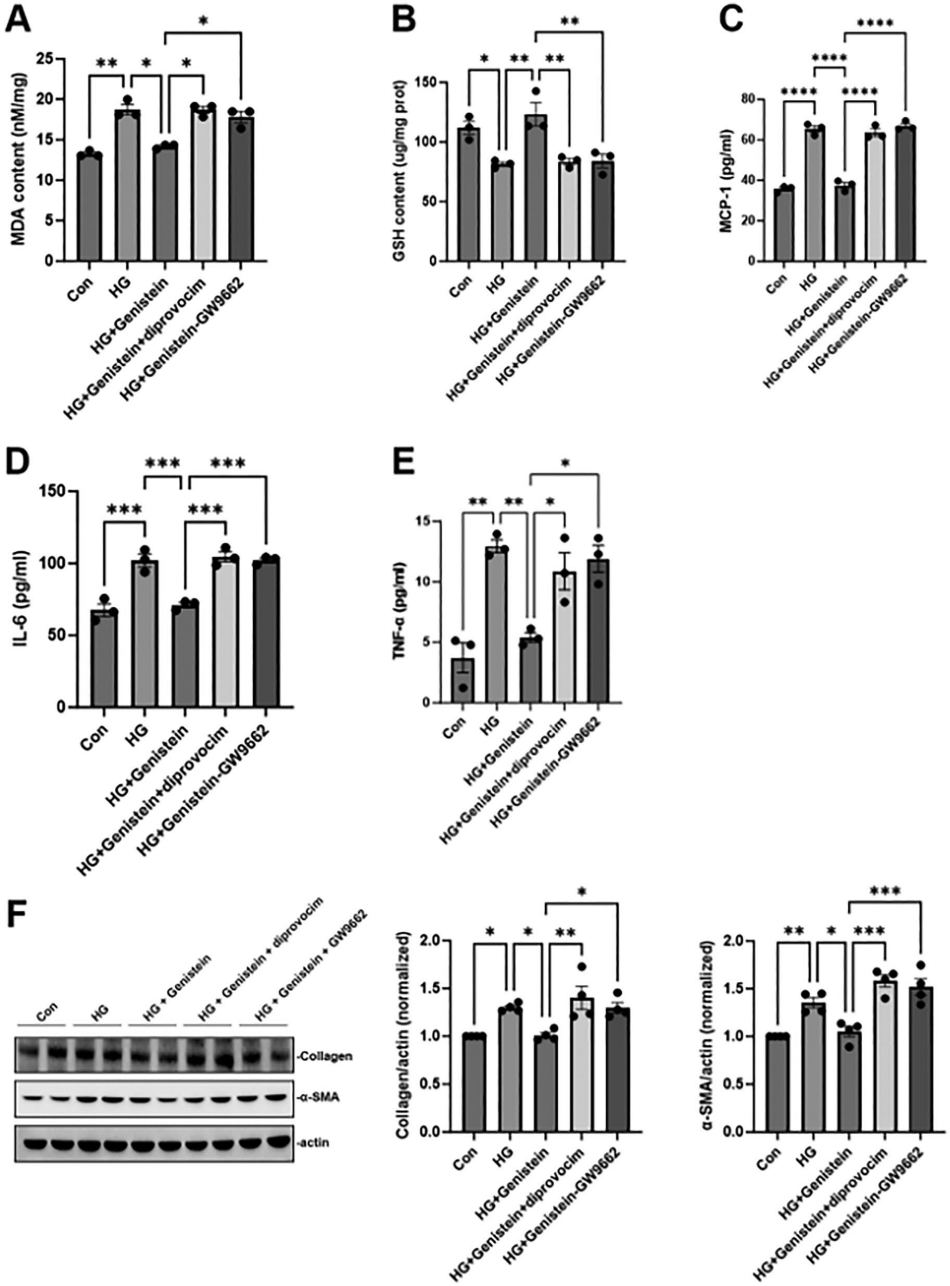
The MAPK/PPAR-γ pathway mediates HG-stimulated EMT in HK-2 cells. (A, B) The concentrations of MDA and GSH were detected in each groups, *n* = 3. (C–E) The MCP-1, IL-6, TNF-α were detected in each groups, *n* = 3. (F) Western blot was performed to analyze the expression of α-SMA and collagen in each group, *n* = 4. Data are presented as mean ± SEM. Asterisks indicate significant differences: * *p* < 0.05.

Previous studies have indicated that the MAPK and PPAR signaling pathways are involved in metabolic regulation. Notably, the p38 MAPK subfamily, a key component of the MAPK pathway, plays a central role in the fibrosis process following inflammatory injury [5,24]. To investigate the functional crosstalk between these pathways and the potential modulatory role of soy protein, we enhanced p38 MAPK expression using diprovocim and inhibited PPARγ with GW9662. Under HG conditions, both the HG + genistein + diprovocim and HG + genistein + GW9662 treatments increased intracellular MDA levels (*n* = 3, Figure 5A), while GSH levels were reduced (*n* = 3, Figure 5B). In addition, the levels of MCP-1, IL-6, and TNF-α in the cell supernatant were upregulated in these treatment groups (*n* = 3, Figure 5C–E). Western blot analysis further revealed increased expression of α-SMA and collagen in both intervention groups compared to the HG group (*n* = 4, Figure 5F and Figure S3). These results suggest that the MAPK/PPAR-γ pathway regulates HG-stimulated EMT in HK-2 cells, and that genistein attenuates this effect.

## 4. Discussion

DKD remains a major complication of T2D, with limited therapeutic options targeting its underlying inflammatory and metabolic pathogenesis [1,25]. The present study systematically investigated the renoprotective effects of soy protein and its key component genistein in DKD, and delineated the mediating role of the MAPKs/PPAR-γ signaling pathway through integrated bioinformatics, *in vivo*, and *in vitro* analyses. The findings collectively support the hypothesis that soy-derived compounds alleviate DKD by restraining inflammation and oxidative stress *via* modulation of the MAPKs/PPAR-γ axis, providing novel mechanistic insights and potential therapeutic implications.

Bioinformatics mining of GEO datasets (GSE154881 and GSE139317) laid the foundation for understanding the molecular landscape of DKD. In GSE154881, comparative analysis of peripheral blood samples from healthy controls, T2D patients, and DKD patients identified 580 DEGs (395 upregulated, 185 downregulated). Functional enrichment analyses revealed that these DEGs were significantly associated with BPs related to inflammation-such as chemotaxis, defense response to Gram-negative bacteria, and regulation of interleukin production-and KEGG pathways including the MAPK signaling pathway. This aligns with prior reports that chronic inflammation is a central driver of DKD progression, as pro-inflammatory cytokines and MAPK-mediated signaling cascades amplify kidney tissue damage [26–28]. The top hub genes identified *via* PPI network analysis-including CCR5, CX3CR1, CCR2, and IL6 are well-characterized regulators of immune cell recruitment and inflammatory responses, further emphasizing the role of inflammation in DKD pathogenesis.

Complementary analysis of GSE139317 (kidney tissues from DKD and sham mice) identified 2830 DEGs (1132 upregulated, 1698 downregulated), with enrichment in fatty acid metabolic processes (e.g. lauric acid and linoleic acid metabolism) and the PPAR signaling pathway. PPAR-γ, a key transcription factor in the PPAR family, is known to regulate lipid homeostasis and suppress inflammation in the kidney; its dysregulation has been linked to impaired kidney function in DKD. The enrichment of PPAR signaling in DKD kidney tissues, alongside the MAPK pathway in blood-derived DEGs, strongly suggested that crosstalk between these two pathways may be critical in DKD. These bioinformatics findings guided subsequent experimental designs, focusing on validating the MAPKs/PPAR-γ axis as a target of soy protein.

In the STZ-induced DKD mouse model, soy protein supplementation exerted dose- and time-dependent renoprotective effects. Biochemical analyses showed that soy protein reversed DKD-associated increases in Scr, BUN, and blood glucose-key markers of kidney dysfunction and hyperglycemia. Histopathological examination further confirmed that soy protein mitigated tubular injury, including epithelial cell shedding, brush border loss, and interstitial fibrosis, as evidenced by H&E and PAS staining. These findings are consistent with previous studies demonstrating the renoprotective effects of soy protein or soy-based diets in DKD animal models and patients. For instance, Kamila et al. reported that isoflavone supplementation improved plasma urea, plasma urea/creatinine ratio, glycemia, water intake, and kidney mass, morphology, and function in type 2 diabetic rats [29]. Similarly, Azadbakht et al. showed that, compared with an animal protein diet, a soy protein diet significantly reduced total cholesterol, triglycerides, LDL-C, urinary urea nitrogen, and proteinuria, while also exerting favorable effects on kidney function in patients with diabetic nephropathy [30]. Beyond confirming these prior observations, our study extends the current knowledge by demonstrating a dose-dependent renoprotective effect of soy protein and linking these improvements to reduced levels of pro-inflammatory cytokines (MCP-1, IL-6, TNF-α) in kidney tissue.

Notably, soy protein also reversed DKD-induced weight loss, a common complication of advanced diabetes associated with catabolic metabolism and renal cachexia [31]. This suggests that soy protein may exert systemic beneficial effects beyond kidney protection, potentially by improving insulin sensitivity or nutrient utilization-though this requires further investigation. Collectively, the *in vivo* data confirm that soy protein ameliorates core features of DKD, including metabolic dysregulation, kidney structural damage, and inflammation.

To dissect the molecular mechanisms underlying soy protein’s effects, we focused on genistein-a major isoflavone in soy with established anti-inflammatory and antioxidant properties [32–34]. In HG-stimulated HK-2 cells, genistein restored cell viability, reduced MDA levels (a marker of oxidative stress), and increased GSH levels (a key antioxidant). These findings indicate that genistein counteracts HG-induced oxidative damage, a critical driver of tubular cell death and fibrosis in DKD.

Consistent with the *in vivo* results, genistein also reduced HG-induced secretion of pro-inflammatory cytokines (MCP-1, IL-6, TNF-α) in a dose-dependent manner. MCP-1, in particular, plays a central role in recruiting monocytes/macrophages to the kidney, amplifying interstitial inflammation and fibrosis. The reduction in these cytokines by genistein suggests that soy protein’s anti-inflammatory effects are, at least in part, mediated by its isoflavone components. Together, these *in vitro* data demonstrate that genistein protects against HG-induced tubular cell injury by targeting both oxidative stress and inflammation.

A critical objective of this study was to validate the role of the MAPKs/PPAR-γ pathway in mediating soy protein/genistein’s effects. Pharmacological manipulation of this pathway in HG-stimulated HK-2 cells revealed that: (1) Diprovocim (a p38 MAPK agonist) reversed genistein’s protective effects, increasing MDA levels, reducing GSH levels, and elevating pro-inflammatory cytokines; (2) GW9662 (a PPAR-γ antagonist) similarly abrogated genistein’s benefits, with concurrent increases in α-SMA and collagen expression-markers of EMT, a key process in renal fibrosis.

The observation that activating p38 MAPK or inhibiting PPAR-γ blocks genistein’s effects directly links the MAPKs/PPAR-γ pathway to the therapeutic potential of soy-derived compounds in DKD. Building on these mechanistic insights, several future directions and clinical implications merit consideration, alongside a clear acknowledgment of current limitations.

Several limitations and future directions should be addressed. First, the *in vivo* experiments used a single DKD model (STZ-induced); validating findings in other models (e.g. db/db mice, which mimic obesity-associated T2D) would strengthen generalizability. Second, DKD is a highly heterogeneous disease with variable clinical trajectories and underlying molecular drivers. Our current findings, derived from a single animal model and a limited set of human datasets, do not fully account for this heterogeneity. Future studies should include diverse DKD subtypes and multi-center human cohorts with longitudinal follow-up to evaluate whether the observed protective effects translate across the full spectrum of disease heterogeneity. Third, while the present study identifies p38 MAPK as a key mediator, other MAPK subfamilies-namely JNK and ERK-have been implicated in DKD pathogenesis and may interact with PPAR-γ [35,36]. Future investigations employing selective inhibitors or gene silencing are warranted to determine whether genistein modulates these pathways and whether such modulation contributes to its renoprotective effects. Fourth, the dose-dependent anti-inflammatory and antioxidant actions observed *in vitro* and *in vivo* support the translational potential of genistein or soy protein as an adjunctive dietary strategy for DKD. Before clinical implementation, rigorous dose-finding studies and long-term safety evaluations in preclinical models are necessary to define the optimal therapeutic window and monitor for potential adverse effects, such as endocrine disruption associated with high-dose isoflavones. Fifth, because genistein is only one of several bioactive components in soy, future studies should systematically compare purified genistein versus whole soy protein, as well as investigate potential synergistic effects with other isoflavones (e.g. daidzein, glycitein) or soy-derived peptides [37,38]. Sixth, from a clinical perspective, randomized controlled trials in DKD patients are needed to evaluate whether soy protein supplementation, alone or combined with standard therapies (e.g. RAAS blockers or SGLT2 inhibitors), reduces hard kidney endpoints such as doubling of serum creatinine, progression to kidney failure, or kidney mortality. Such trials should also assess patient-reported outcomes and cost-effectiveness.

## 5. Conclusion

In summary, this study demonstrates that soy protein alleviates DKD by restraining inflammation and oxidative stress, with genistein acting as a key mediator. The underlying mechanism involves modulation of the MAPKs/PPAR-γ signaling pathway: soy-derived genistein inhibits p38 MAPK-mediated inflammation and activates PPAR-γ-dependent anti-inflammatory and antioxidant responses, thereby mitigating tubular injury and fibrosis. These findings validate the MAPKs/PPAR-γ axis as a therapeutic target in DKD and support the potential of soy-based interventions as a complementary strategy for DKD management.

## Supplementary Material

Supplemental Material

Supplemental Material

Supplemental Material

## Data Availability

The authors confirm that the data supporting the findings of this study are available within the article and its supplementary materials, and have also been deposited in the Science Data Bank under the Data CSTR identifier 31253.11. sciencedb.37504 and the DOI: 10.57760/sciencedb.37504.
