# Supplementary material for: Soy protein alleviates DKD by restraining inflammation via the MAPKs/PPAR-γ signaling pathway
Source: Ren Fail. 2026 Jul 24;48(1):2698775. doi: 10.1080/0886022X.2026.2698775 (PMC13403453; doi:10.1080/0886022X.2026.2698775)

Supplementary Figure 1 (Figure S1). Representative original HE-stained images of mouse kidney sections in each group.


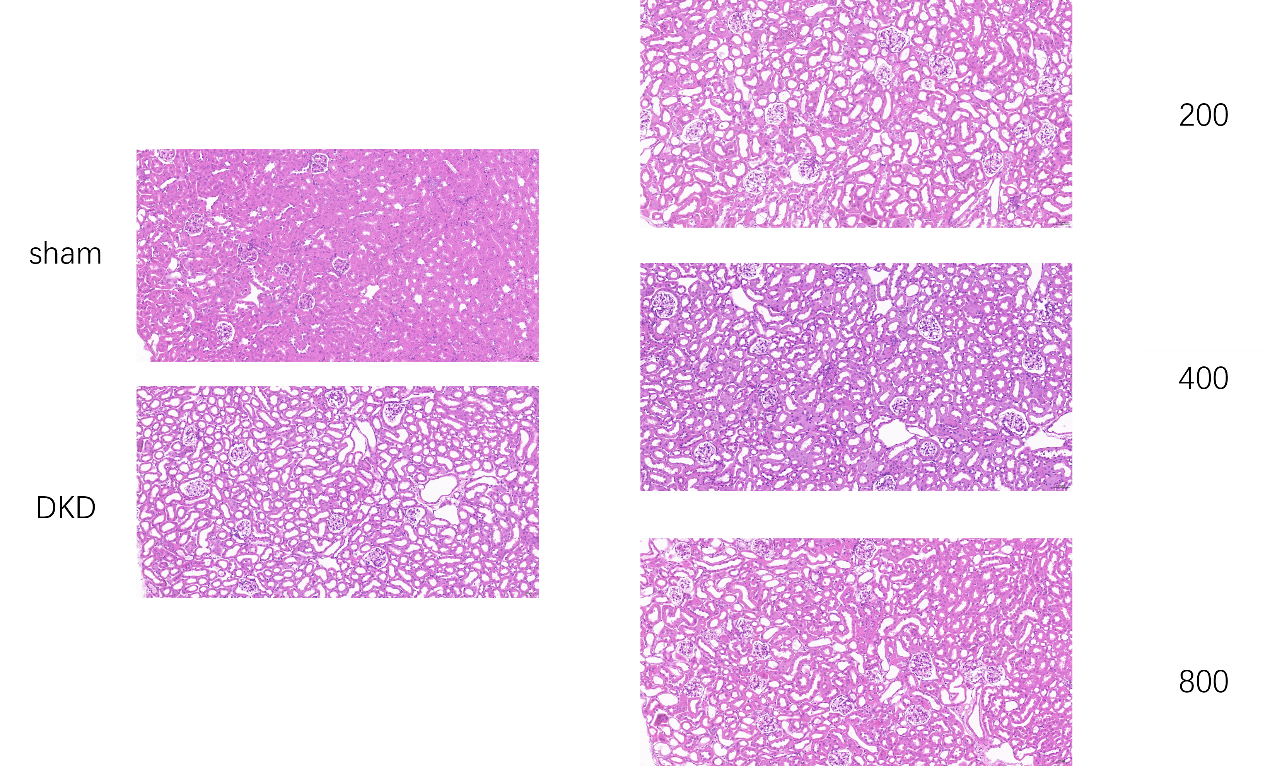


Supplementary Figure 2 (Figure S2). Representative original PAS-stained images of mouse kidney sections in each group.


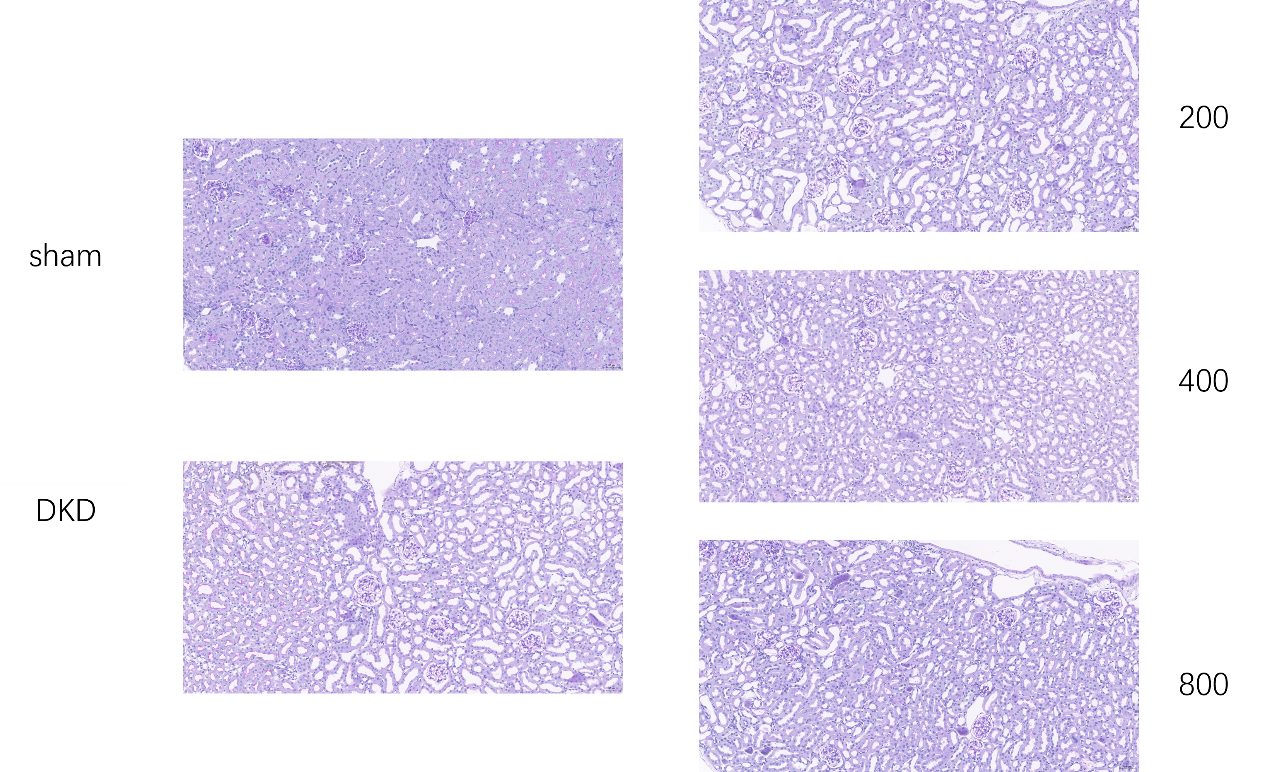


Supplementary Figure 3 (Figure S3). Representative original Western blot results showing α-SMA and collagen expression in each group.


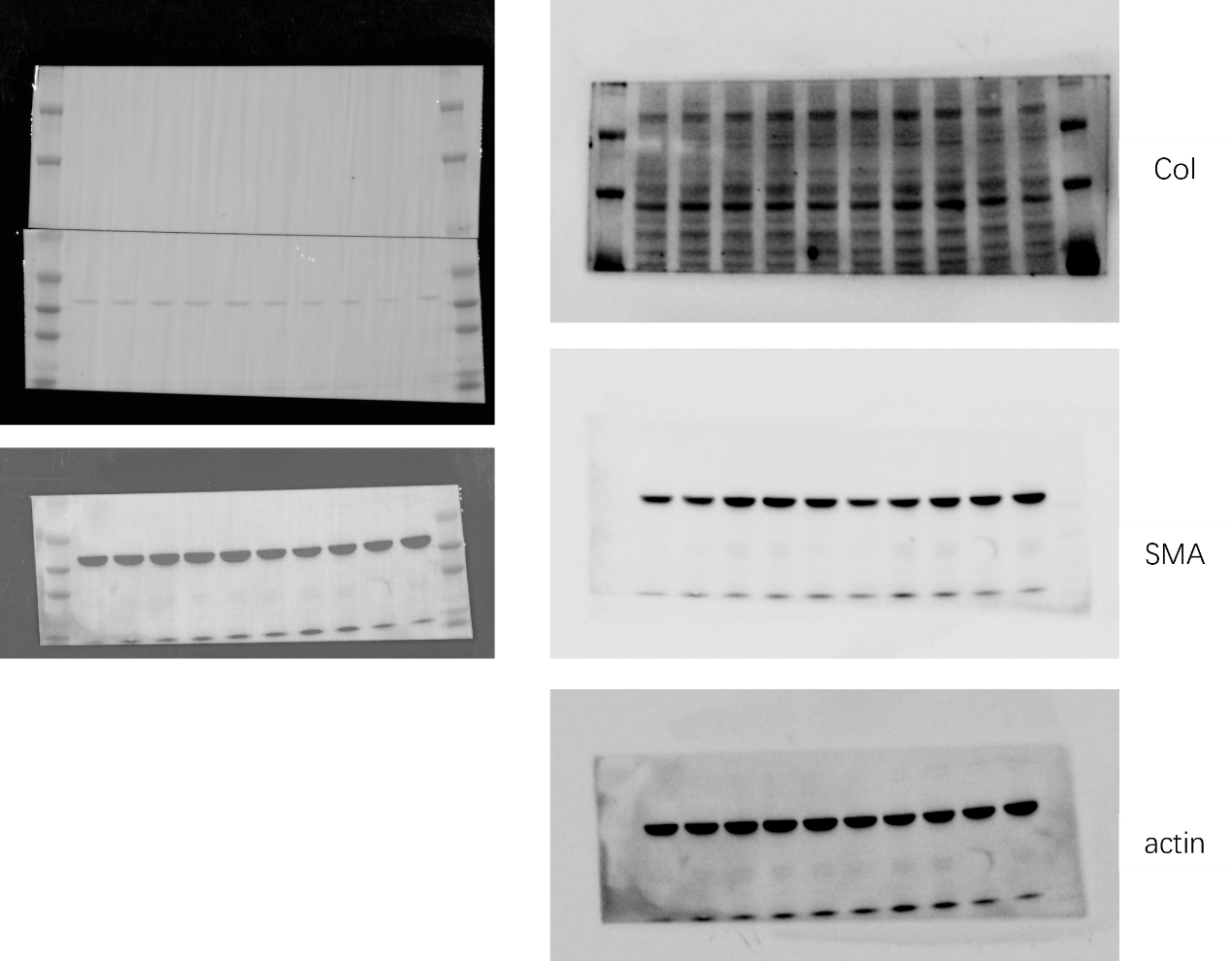

Supplement: Supplemental Material [file IRNF_A_2698775_SM2688.docx]
