# Supplementary material for: Soy protein alleviates DKD by restraining inflammation via the MAPKs/PPAR-γ signaling pathway
Source: Ren Fail. 2026 Jul 24;48(1):2698775. doi: 10.1080/0886022X.2026.2698775 (PMC13403453; doi:10.1080/0886022X.2026.2698775)
Supplement: Supplemental Material [file IRNF_A_2698775_SM2508.docx]

**Supplementary table 1.** Functional and pathway enrichment analyses of differentially expressed genes in the GSE154881 dataset.

| Term | Count | PValue | Genes |
| --- | --- | --- | --- |
| hsa05134Legionellosis | 8 | 5.08E-05 | NFKBIA, IL6, HSPA1L, HSPA6, NLRC4, TLR4, HSPA1B, HSPA1A |
| hsa01240Biosynthesis of cofactors | 12 | 7.98E-05 | VKORC1L1, COQ3, UGDH, MAT2A, NMNAT1, QPRT, PNPO, MAT2B, KMO, BCAT1, EARS2, IDO1 |
| GO0006935~chemotaxis | 10 | 3.56E-04 | SPN, CCR1, RIPOR2, CXCR2, MOSPD2, DEFA1, CXCR6, DEFA1B, CCR3, CMKLR1 |
| hsa04061Viral protein interaction with cytokine and cytokine receptor | 9 | 3.59E-04 | CCR1, CSF1R, IL6, CXCR2, TNFSF10, TNFRSF10C, IL6R, CCR3, TNFRSF1A |
| GO0050829~defense response to Gram-negative bacterium | 9 | 3.67E-04 | IL6, IL23A, NFKBIZ, BPI, DEFA1, DEFA1B, IL6R, TLR4, LTF |
| hsa04621NOD-like receptor signaling pathway | 12 | 4.81E-04 | NFKBIA, IL6, RNASEL, NLRP12, IFI16, NEK7, CARD6, DEFA1, NLRC4, DEFA1B, ANTXR2, TLR4 |
| GO0032757~positive regulation of interleukin-8 production | 7 | 8.06E-04 | IL6, F2RL1, TLR8, TLR4, HSPA1B, RIGI, HSPA1A |
| GO0032731~positive regulation of interleukin-1 beta production | 7 | 8.74E-04 | IL6, NLRP12, IFI16, F2RL1, TLR8, NLRC4, TLR4 |
| GO0046872~metal ion binding | 71 | 0.001089 | DPAGT1, RTP4, MOCS3, STEAP4, ZNF691, CISD1, SLC40A1, ABAT, ANTXR2, LFNG, ZNF607, ZNF41, TNFSF10, ME3, TRIM27, ZNF818P, ZNF566, AGFG2, ZNF486, ZNF200, KLF10, NSF, ZC3H10, ZSCAN5A, ZFP3, IPMK, RUFY1, ISL2, MBLAC2, TRAF4, ZNF559, ZNF780B, ZNF799, FAM20A, GART, TP53, ZNF234, IDO1, ZNF28, ZNF397, CSF1R, ZNF396, RNASEL, CASZ1, ZNF790, RPE, ZBTB3, PPP2CB, ZNF137P, HMGCL, MAT2A, RSBN1L, HLTF, ZKSCAN3, BPNT1, ZNF546, ZNF226, ZNF786, ZNF585B, ZNF420, ZNF584, IDH1, NEK7, ZBTB10, IRF2BP2, B4GAT1, PUDP, VPS41, STT3A, ZNF696, ZNF772 |
| GO0140911~pore-forming activity | 3 | 0.001667 | PRF1, DEFA1, DEFA1B |
| GO0051085~chaperone cofactor-dependent protein refolding | 5 | 0.002001 | HSPA1L, HSPA6, DNAJB13, HSPA1B, HSPA1A |
| GO0042802~identical protein binding | 47 | 0.002352 | DPAGT1, GPSM2, RIPOR2, RNASEL, TAMALIN, PRAG1, HVCN1, RPE, SDC2, QPRT, NAB2, PDGFB, CISD1, SLC40A1, PRF1, ABAT, ADRB2, NLRC4, TCL1A, IFI16, MAT2A, KCTD21, LMNA, TNFSF10, TRIM27, IER5, SANBR, CASP8AP2, WHRN, IDH1, RUFY1, NFKBIA, UGDH, NIF3L1, IL6, TRAF4, NMNAT1, VPS41, LCN2, TLR8, TLR10, PTX3, LY6G5B, ESYT1, TP53, TLR4, RIGI |
| GO0051607~defense response to virus | 12 | 0.002792 | RTP4, IL6, RNASEL, IFI16, IL23A, IRF2, PRF1, F2RL1, TLR8, DEFA1, DEFA1B, RIGI |
| GO0050729~positive regulation of inflammatory response | 8 | 0.002986 | NFKBIA, NLRP12, IL23A, NFKBIZ, MIR22, NLRC4, TLR4, TNFRSF1A |
| GO0006954~inflammatory response | 17 | 0.003011 | CCR1, CSF1R, CXCR6, NLRC4, TNFRSF1A, IL6, IFI16, IL23A, CXCR2, F2RL1, TLR8, TLR10, PTX3, TLR4, CCR3, IDO1, CMKLR1 |
| hsa05162Measles | 9 | 0.003058 | NFKBIA, IL6, HSPA1L, HSPA6, TP53, TLR4, HSPA1B, RIGI, HSPA1A |
| GO0007204~positive regulation of cytosolic calcium ion concentration | 9 | 0.00307 | CCR1, CXCR2, LPAR1, F2RL1, CHRNA10, CXCR6, GATA2, CCR3, CMKLR1 |
| GO0005515~protein binding | 254 | 0.003465 | JRK, JPT2, PRF1, ANTXR2, DCAF7, SMC2, C1GALT1C1, TCL1A, ZNF607, TNFSF10, CYP1B1, SMCO4, TRIM27, IER5, ANKS1B, CMKLR1, ARL11, MEF2C, ZFP3, GOLPH3L, KNSTRN, PISD, WDR77, RUFY1, NIF3L1, ISL2, IL23A, HECW2, PPP1R3D, SIK1, TP53, ZNF397, ZNF396, ABCB1, SDC2, PDGFB, LPAR1, TMTC1, C9ORF78, GATA2, GSPT2, ZBTB3, HLTF, PNPO, SLAMF6, ATP6V1C1, CCR1, JUND, ICMT, FZD2, SH2D1B, RMDN2, VASN, H1-3, SULT1B1, H1-4, B4GAT1, SFXN2, NDUFAF1, CIBAR1, MRS2, MOCS3, DIRAS1, QPRT, SHB, PIGV, TMEM186, MS4A4A, SHE, NHSL2, ADAMTSL5, NUDCD1, ZNF41, TNFAIP8L2, CCR3, IKBIP, TIGD3, PRMT6, DUSP5, ZC3H10, ZSCAN5A, NOG, MYOF, EMP1, MLF1, MN1, MBLAC2, RBM12B, MED20, CDC42EP2, TLR8, TLR10, PIGM, FAM20A, TLR4, PCYOX1, DYNC1I2, RNASEL, SELPLG, SAMD9, FAAP24, FSTL1, PURA, PPP2CB, SERTAD2, HSD17B1, LMNA, CXCR2, METTL6, EXOSC3, MPZL2, P2RY13, CASP8AP2, TRMT10A, UTP6, GABBR1, HSPA1L, WHRN, YIPF4, CARD6, NLRP12, NMNAT1, ETNK2, F2RL1, HYLS1, ESYT1, KBTBD7, ZNF696, HSPA1B, HSPA1A, TMEM229B, DPAGT1, RIPOR2, VKORC1L1, SAMD9L, ZNF691, PRAG1, CEP19, PROS1, NAB2, GIMAP1, WBP1L, GIMAP5, LOXL1, CHCHD4, CFL2, ZNF566, IL6R, ZNF200, EPM2A, NSF, GUCY1A1, ANKRD46, IPMK, IRAG1, DNAJB13, TMEM250, KMO, CLUHP3, SCNN1D, MSH2, ZNF559, SBDS, HOMEZ, CSF1R, KCNE3, KLHL15, HVCN1, ABHD6, ADRB2, CSF2RA, ZKSCAN3, ZNF786, ZNF420, CBX8, COA3, IDH1, CPSF2, HSPA6, NEK7, TNFRSF10C, ZBTB10, DEFA1, ARPC5, NFKBIA, COQ3, DAB2, IL6, RGCC, TFCP2, NAT1, PUDP, STT3A, LCN2, NFE2L3, MANSC1, RIGI, ZNF772, RTP4, GPSM2, COLGALT2, KCNK7, NXT1, SLC40A1, DNAAF10, SPN, METTL13, NFKBIZ, PGM2, ACP2, ARHGEF11, SMIM14, KLF10, PVRIG, C14ORF119, DARS2, TNFRSF1A, ETV7, PEAK3, MMRN1, TRAF4, TRAPPC6B, IRF2, MCM3, ATG4C, BCAT1, LTF, PPP1R15A, PPP1R15B, PARG, RPE, FGL2, MOSPD2, NLRC4, MAT2B, MRM1, PRRG4, MAT2A, IFI16, KCTD21, TP53INP2, EEF2KMT, NRIP3, NUP43, DEFA1B, SCIMP, MFSD14B, IRGQ, CLCN4, KATNBL1, COL7A1, VPS41, PTX3, MNDA |
| GO0070434~positive regulation of nucleotide-binding oligomerization domain containing 2 signaling pathway | 3 | 0.004104 | TLR4, HSPA1B, HSPA1A |
| GO0072126~positive regulation of glomerular mesangial cell proliferation | 3 | 0.004104 | PDGFD, PDGFB, IL6R |
| GO0045087~innate immune response | 20 | 0.004111 | CSF1R, SAMD9, HVCN1, SH2D1B, NLRC4, IFI16, TRAF4, IL23A, LCN2, F2RL1, TLR8, TLR10, PTX3, SLAMF6, BPI, TRIM27, TNFAIP8L2, TLR4, RIGI, LTF |
| GO0045190~isotype switching | 4 | 0.004436 | SANBR, MSH2, NFKBIZ, EXOSC3 |
| GO1904813~ficolin-1-rich granule lumen | 8 | 0.005187 | IDH1, FGL2, HSPA6, PGM2, MNDA, ARPC5, HSPA1B, HSPA1A |
| GO0140545~ATP-dependent protein disaggregase activity | 3 | 0.00564 | NSF, HSPA1B, HSPA1A |
| hsa04060Cytokine-cytokine receptor interaction | 13 | 0.006166 | CCR1, CSF1R, TNFRSF10C, CXCR6, CSF2RA, TNFRSF1A, IL6, IL23A, CXCR2, TNFSF10, TNFRSF8, IL6R, CCR3 |
| GO0019957~C-C chemokine binding | 4 | 0.006571 | CCR1, CXCR2, CXCR6, CCR3 |
| GO0016493~C-C chemokine receptor activity | 4 | 0.006571 | CCR1, CXCR2, CXCR6, CCR3 |
| GO0032722~positive regulation of chemokine production | 5 | 0.00698 | CSF1R, IL6, F2RL1, IL6R, TLR4 |
| GO0032755~positive regulation of interleukin-6 production | 7 | 0.007813 | IL6, F2RL1, TLR8, SCIMP, IL6R, TLR4, RIGI |
| GO0000981~DNA-binding transcription factor activity, RNA polymerase II-specific | 34 | 0.008002 | ZNF397, ZNF28, HOMEZ, ZNF396, CASZ1, ZNF790, GATA2, ZBTB3, ZNF137P, PURA, ZNF607, ZNF41, ZKSCAN3, ZNF546, ZNF566, ZNF420, ZNF486, KLF10, ZNF584, ZSCAN5A, MEF2C, JUND, ZFP3, PROX2, ETV7, ISL2, TFCP2, IRF2, NFE2L3, ZNF559, ZNF799, TP53, ZNF696, ZNF772 |
| hsa05202Transcriptional misregulation in cancer | 10 | 0.00809 | ETV7, CSF1R, MEF2C, IL6, SLC45A3, NFKBIZ, DEFA1, DEFA1B, TP53, MLF1 |
| GO0002548~monocyte chemotaxis | 4 | 0.008408 | CCR1, IL6, PDGFB, IL6R |
| GO0006357~regulation of transcription by RNA polymerase II | 43 | 0.009517 | ZNF397, ZNF28, HOMEZ, ZNF396, ZNF691, ZNF790, GATA2, ZBTB3, PPP2CB, ZNF137P, PURA, ZNF607, ZNF41, ZKSCAN3, ZNF546, ZNF226, ZNF786, ZNF585B, ZNF566, ZNF420, ZNF486, EPM2A, KLF10, ZNF584, DUSP5, ZSCAN5A, JUND, ZFP3, ZBTB10, IRF2BP2, PROX2, WDR77, ETV7, TFCP2, MED20, IRF2, NFE2L3, ZNF559, ZNF799, ZNF780B, TP53, ZNF696, ZNF772 |
| GO0042026~protein refolding | 4 | 0.010431 | HSPA1L, HSPA6, HSPA1B, HSPA1A |
| GO0002224~toll-like receptor signaling pathway | 4 | 0.010431 | NFKBIZ, TLR8, TLR10, TLR4 |
| GO0032729~positive regulation of type II interferon production | 6 | 0.010437 | IL23A, F2RL1, TLR8, SLAMF6, TRIM27, TLR4 |
| hsa05164Influenza A | 9 | 0.011154 | NFKBIA, IL6, RNASEL, NXT1, TNFSF10, TLR4, RIGI, PABPN1L, TNFRSF1A |
| GO0003690~double-stranded DNA binding | 7 | 0.011396 | H1-4, JUND, MSH2, IFI16, MNDA, H1-3, RIGI |
| GO0090023~positive regulation of neutrophil chemotaxis | 4 | 0.011538 | RIPOR2, IL23A, CXCR2, MOSPD2 |
| GO0071260~cellular response to mechanical stimulus | 6 | 0.011567 | RIPOR2, CASP8AP2, TLR8, TNFRSF8, TLR4, TNFRSF1A |
| GO0045669~positive regulation of osteoblast differentiation | 6 | 0.011567 | MEF2C, IL6, MIR3648-1, JUND, IL6R, LTF |
| GO0072540~T-helper 17 cell lineage commitment | 3 | 0.011771 | IL6, SLAMF6, IL6R |
| GO0048661~positive regulation of smooth muscle cell proliferation | 5 | 0.013162 | IL6, PDGFD, PDGFB, IL6R, TLR4 |
| hsa05417Lipid and atherosclerosis | 10 | 0.013798 | NFKBIA, IL6, HSPA1L, HSPA6, TNFSF10, TP53, TLR4, HSPA1B, HSPA1A, TNFRSF1A |
| hsa05145Toxoplasmosis | 7 | 0.013888 | NFKBIA, HSPA1L, HSPA6, TLR4, HSPA1B, HSPA1A, TNFRSF1A |
| GO0038023~signaling receptor activity | 10 | 0.015704 | GUCY1A1, PVRIG, F2RL1, TLR8, TLR10, ANTXR2, TLR4, CSF2RA, CMKLR1, TNFRSF1A |
| GO0006968~cellular defense response | 5 | 0.015807 | SPN, CXCR2, PRF1, MNDA, CCR3 |
| hsa04010MAPK signaling pathway | 12 | 0.016514 | DUSP5, CSF1R, MEF2C, JUND, HSPA1L, PDGFD, HSPA6, PDGFB, TP53, HSPA1B, HSPA1A, TNFRSF1A |
| GO0031072~heat shock protein binding | 5 | 0.019583 | SPN, HSPA1L, HSPA6, HSPA1B, HSPA1A |
| GO1901731~positive regulation of platelet aggregation | 3 | 0.019732 | IL6, MMRN1, IL6R |
| GO0006955~immune response | 17 | 0.021289 | CCR1, IGSF6, DEFA1, CXCR6, SLED1, TRAV29DV5, SPN, IL6, CXCR2, TNFSF10, TRAV14DV4, TLR10, SLAMF6, DEFA1B, TLR4, CCR3, CMKLR1 |
| GO0042826~histone deacetylase binding | 7 | 0.021587 | H1-4, MEF2C, KCTD21, SIK1, TP53, HSPA1B, HSPA1A |
| GO0000164~protein phosphatase type 1 complex | 3 | 0.022013 | PPP1R15A, PPP1R15B, PPP1R3D |
| GO0002690~positive regulation of leukocyte chemotaxis | 3 | 0.022766 | IL6, F2RL1, IL6R |
| GO0051260~protein homooligomerization | 7 | 0.025057 | KCNG1, RIPOR2, KCTD21, MOSPD2, PRF1, NLRC4, KCTD3 |
| GO0034058~endosomal vesicle fusion | 3 | 0.025979 | RUFY1, SAMD9, VPS41 |
| GO0032682~negative regulation of chemokine production | 3 | 0.025979 | IL6, F2RL1, MIR590 |
| hsa05130Pathogenic Escherichia coli infection | 9 | 0.026565 | ARHGEF11, NFKBIA, IL6, LPAR5, TNFSF10, LPAR1, ARPC5, TLR4, TNFRSF1A |
| GO0070098~chemokine-mediated signaling pathway | 5 | 0.030977 | CCR1, CXCR2, CXCR6, CCR3, CMKLR1 |
| GO0045910~negative regulation of DNA recombination | 3 | 0.032912 | H1-4, MSH2, H1-3 |
| GO2000573~positive regulation of DNA biosynthetic process | 3 | 0.032912 | RGCC, PDGFB, CYP1B1 |
| GO0002384~hepatic immune response | 2 | 0.033598 | IL6, IL6R |
| GO0044793~negative regulation by host of viral process | 2 | 0.033598 | PTX3, LTF |
| GO0034276~kynurenic acid biosynthetic process | 2 | 0.033598 | KMO, IDO1 |
| GO0032733~positive regulation of interleukin-10 production | 4 | 0.034022 | IL6, IL23A, F2RL1, TLR4 |
| GO0019221~cytokine-mediated signaling pathway | 7 | 0.034122 | CCR1, CSF1R, IL6, NFKBIZ, IL6R, CSF2RA, TNFRSF1A |
| GO0140662~ATP-dependent protein folding chaperone | 4 | 0.035797 | HSPA1L, HSPA6, HSPA1B, HSPA1A |
| GO0000030~mannosyltransferase activity | 3 | 0.036375 | TMTC1, PIGM, PIGV |
| GO0010165~response to X-ray | 3 | 0.036619 | IKBIP, MSH2, TP53 |
| GO0072562~blood microparticle | 7 | 0.037627 | IGHG4, HSPA1L, PROS1, HSPA6, CP, HSPA1B, HSPA1A |
| GO0042101~T cell receptor complex | 7 | 0.037627 | TRAV3, TRAV12-2, TRAV14DV4, TRAV21, TRAV8-1, TRAV9-2, TRAV29DV5 |
| GO0032088~negative regulation of NF-kappaB transcription factor activity | 5 | 0.040108 | NFKBIA, PPP2CB, NLRP12, CYP1B1, CMKLR1 |
| GO0019731~antibacterial humoral response | 5 | 0.040108 | IGHG4, BPI, DEFA1, DEFA1B, LTF |
| GO0004950~chemokine receptor activity | 3 | 0.04021 | CCR1, CCR3, CMKLR1 |
| GO0031396~regulation of protein ubiquitination | 3 | 0.040478 | EPM2A, HSPA1B, HSPA1A |
| GO0032735~positive regulation of interleukin-12 production | 4 | 0.040538 | IL23A, SCIMP, TLR4, IDO1 |
| GO0032760~positive regulation of tumor necrosis factor production | 6 | 0.041944 | SPN, IL6, IL23A, TNFRSF8, TLR4, RIGI |
| GO0000978~RNA polymerase II cis-regulatory region sequence-specific DNA binding | 29 | 0.042216 | ZNF397, ZNF28, ZNF396, ZNF691, ZNF790, GATA2, ZNF137P, ZNF607, NFKBIZ, ZNF41, ZKSCAN3, ZNF546, ZNF226, ZNF786, ZNF585B, ZNF420, ZNF486, KLF10, ZNF584, ZSCAN5A, MEF2C, JUND, PROX2, TFCP2, IRF2, NFE2L3, ZNF780B, TP53, ZNF772 |
| hsa01230Biosynthesis of amino acids | 5 | 0.042665 | MAT2A, RPE, IDH1, MAT2B, BCAT1 |
| GO0004888~transmembrane signaling receptor activity | 8 | 0.044859 | SPN, IGSF6, TNFRSF10C, CHRNA10, TNFRSF8, TLR10, ANTXR2, TLR4 |
| GO0006355~regulation of DNA-templated transcription | 25 | 0.045043 | ZNF28, CASZ1, ZNF790, NAB2, ZNF607, ZNF41, ZNF546, ZNF226, ZNF818P, ZNF585B, ZNF566, ZNF420, ZNF486, PRMT6, ZNF584, MEF2C, PROX2, MLF1, MN1, IRF2, ZNF559, ZNF780B, TP53, ZNF234, ZNF772 |
| GO0005739~mitochondrion | 39 | 0.045057 | PPP1R15A, MRS2, AARS1, SAMD9L, CCDC127, CISD1, ABAT, MRM1, TMEM186, CHCHD4, HMGCL, METTL13, PNPO, CYP1B1, BPNT1, ME3, TRIM27, EARS2, CASP8AP2, COA3, C14ORF119, IDH1, DARS2, RMDN2, KMO, VASN, PISD, COQ3, NIF3L1, MBLAC2, NUDT8, SFXN2, NDUFAF1, BCAT1, CIBAR1, SLC25A33, TP53, HSPA1B, HSPA1A |
| GO0071222~cellular response to lipopolysaccharide | 8 | 0.04534 | MEF2C, IL6, NFKBIZ, BPI, DEFA1, DEFA1B, KMO, TLR4 |
| hsa05163Human cytomegalovirus infection | 9 | 0.045617 | ARHGEF11, NFKBIA, CCR1, IL6, CXCR2, IL6R, TP53, CCR3, TNFRSF1A |
| GO0048545~response to steroid hormone | 3 | 0.048627 | DAB2, JUND, IDH1 |
| GO0014911~positive regulation of smooth muscle cell migration | 3 | 0.048627 | PDGFB, CYP1B1, TLR4 |
| GO0048269~methionine adenosyltransferase complex | 2 | 0.049092 | MAT2A, MAT2B |
| GO0005896~interleukin-6 receptor complex | 2 | 0.049092 | IL6, IL6R |
| GO0070401~NADP+ binding | 2 | 0.049794 | HSD17B1, ME3 |
| GO0004376~glycolipid mannosyltransferase activity | 2 | 0.049794 | PIGM, PIGV |
| hsa01100Metabolic pathways | 37 | 0.049803 | DPAGT1, VKORC1L1, COLGALT2, RPE, QPRT, HSD17B4, ABAT, MAT2B, PIGV, HMGCL, C1GALT1C1, MAT2A, HSD17B1, PNPO, PGM2, BPNT1, ACP2, ME3, ATP6V1C1, EARS2, GUCY1A1, IDH1, IPMK, B3GALT6, KMO, PISD, COQ3, UGDH, NAT1, B4GAT1, NMNAT1, ETNK2, STT3A, PIGM, BCAT1, GART, IDO1 |
| GO0021533~cell differentiation in hindbrain | 2 | 0.049973 | NOG, GATA2 |
| GO1903799~negative regulation of miRNA processing | 2 | 0.049973 | ZC3H10, TP53 |
| GO0003104~positive regulation of glomerular filtration | 2 | 0.049973 | PDGFB, F2RL1 |
| GO0021549~cerebellum development | 4 | 0.052683 | LPAR1, ABAT, TP53, GART |
| GO0030165~PDZ domain binding | 5 | 0.053823 | NSF, TAMALIN, FZD2, SDC2, LPAR1 |
| GO0030261~chromosome condensation | 3 | 0.057311 | H1-4, H1-3, SMC2 |
| GO0000122~negative regulation of transcription by RNA polymerase II | 24 | 0.059859 | PRMT6, CBX8, KLF10, MEF2C, JUND, NOG, ZBTB10, IRF2BP2, GATA2, H1-3, NFKBIA, ETV7, H1-4, PURA, DAB2, NIF3L1, IFI16, IRF2, NFE2L3, ZKSCAN3, TRIM27, LINC-PINT, TP53, HSPA1A |
| GO0060326~cell chemotaxis | 5 | 0.060404 | CCR1, PDGFB, LPAR1, CXCR6, CCR3 |
| GO0006556~S-adenosylmethionine biosynthetic process | 2 | 0.066071 | MAT2A, MAT2B |
| GO1990966~ATP generation from poly-ADP-D-ribose | 2 | 0.066071 | PARG, NMNAT1 |
| GO0098990~AMPA selective glutamate receptor signaling pathway | 2 | 0.066071 | MEF2C, ADRB2 |
| GO0019805~quinolinate biosynthetic process | 2 | 0.066071 | KMO, IDO1 |
| GO0034314~Arp23 complex-mediated actin nucleation | 3 | 0.066487 | TRIM27, WHAMM, ARPC5 |
| GO0036120~cellular response to platelet-derived growth factor stimulus | 3 | 0.066487 | PDGFD, PDGFB, TLR4 |
| GO0051082~unfolded protein binding | 6 | 0.067795 | HSPA1L, HSPA6, DNAJB13, NDUFAF1, HSPA1B, HSPA1A |
| GO0016363~nuclear matrix | 6 | 0.068077 | RNASEL, CFL2, LMNA, HLTF, TP53, DCAF7 |
| GO0008270~zinc ion binding | 47 | 0.068326 | ZNF397, ZNF28, RTP4, ZNF396, AARS1, RNASEL, CASZ1, ZNF691, ZNF790, GATA2, ZBTB3, ZNF607, HLTF, ZNF41, TNFSF10, ZKSCAN3, ZNF546, TRIM6-TRIM34, ZNF226, TRIM27, ZNF786, ZNF818P, ZNF585B, ZNF566, AGFG2, EARS2, ZNF200, ZNF420, ZNF486, KLF10, ZNF584, ZC3H10, ZSCAN5A, ZFP3, ZBTB10, IRF2BP2, RUFY1, TRAF4, VPS41, ZNF559, ZNF799, ZNF780B, TP53, ZNF696, ZNF234, ZNF772, RIGI |
| GO0005634~nucleus | 118 | 0.069076 | JRK, ZNF691, PRAG1, JPT2, NAB2, DCAF7, PABPN1L, SMC2, TCL1A, ZNF607, PARTICL, TRIM27, ZNF566, IER5, ZNF200, EPM2A, MEF2C, ZFP3, IPMK, TMEM250, KNSTRN, PISD, WDR77, NIF3L1, ISL2, GVINP1, MSH2, ZNF559, SBDS, SIK1, ZNF799, TP53, ZNF397, ZNF28, HOMEZ, ZNF396, AARS1, CASZ1, ZNF790, KLHL15, C9ORF78, ADRB2, GATA2, ZBTB3, HLTF, ZKSCAN3, ZNF546, ZNF786, ZNF420, CBX8, JUND, NEK7, HSPA6, ZBTB10, IRF2BP2, ARPC5, H1-3, NFKBIA, H1-4, RGCC, TFCP2, NFE2L3, CIBAR1, ZNF772, NXT1, METTL13, NUDCD1, NFKBIZ, ZNF41, TNFAIP8L2, ZNF818P, ZNF486, PRMT6, KLF10, TIGD3, DUSP5, ZC3H10, ZSCAN5A, PROX2, MLF1, ETV7, UGDH, MN1, TRAF4, MED20, IRF2, MCM3, ZNF780B, ZNF234, LTF, PARG, MAT2B, ZNF137P, PPP2CB, PURA, IFI16, SERTAD2, TP53INP2, RSBN1L, LMNA, NUP43, METTL6, ZNF226, LINC-PINT, EXOSC3, ZNF585B, ZNF584, CASP8AP2, TRMT10A, HSPA1L, NMNAT1, KATNBL1, HYLS1, MNDA, KBTBD7, ZNF696, HSPA1B, HSPA1A |
| GO0034605~cellular response to heat | 4 | 0.069282 | HSPA6, IER5, HSPA1B, HSPA1A |
| GO0000977~RNA polymerase II transcription regulatory region sequence-specific DNA binding | 11 | 0.070621 | ETV7, PURA, MEF2C, CASZ1, ZFP3, IRF2, ZBTB10, ZNF559, ZNF799, ZNF566, ZNF696 |
| GO1901673~regulation of mitotic spindle assembly | 3 | 0.071245 | RIPOR2, HSPA1B, HSPA1A |
| GO0043235~receptor complex | 8 | 0.071994 | CSF1R, TLR10, ADRB2, SCIMP, IL6R, TLR4, CSF2RA, TNFRSF1A |
| GO0031625~ubiquitin protein ligase binding | 10 | 0.07451 | NFKBIA, ABCB1, TRAF4, HSPA1L, HLTF, WBP1L, TP53, HSPA1B, RIGI, HSPA1A |
| hsa05132Salmonella infection | 9 | 0.074684 | NFKBIA, DYNC1I2, IL6, VPS41, TNFSF10, NLRC4, ARPC5, TLR4, TNFRSF1A |
| GO0007259~cell surface receptor signaling pathway via JAK-STAT | 4 | 0.075279 | IL6, IL23A, CSF2RA, TNFRSF1A |
| GO0097696~cell surface receptor signaling pathway via STAT | 3 | 0.07611 | IL6, IL23A, IL6R |
| GO0032740~positive regulation of interleukin-17 production | 3 | 0.07611 | IL6, IL23A, SLAMF6 |
| GO0032727~positive regulation of interferon-alpha production | 3 | 0.07611 | TLR8, TLR4, RIGI |
| GO0050853~B cell receptor signaling pathway | 4 | 0.078362 | NFKBIA, IGHG4, MEF2C, MNDA |
| GO0044183~protein folding chaperone | 4 | 0.08081 | HSPA1L, HSPA6, HSPA1B, HSPA1A |
| GO0055131~C3HC4-type RING finger domain binding | 2 | 0.081609 | HSPA1B, HSPA1A |
| GO0045569~TRAIL binding | 2 | 0.081609 | TNFSF10, TNFRSF10C |
| GO0070427~nucleotide-binding oligomerization domain containing 1 signaling pathway | 2 | 0.081897 | NFKBIA, TLR4 |
| GO0071673~positive regulation of smooth muscle cell chemotaxis | 2 | 0.081897 | PDGFD, LPAR1 |
| GO0070370~cellular heat acclimation | 2 | 0.081897 | HSPA1B, HSPA1A |
| GO0032259~methylation | 7 | 0.084116 | PRMT6, TRMT10A, COQ3, METTL13, ICMT, EEF2KMT, METTL6 |
| GO1902895~positive regulation of miRNA transcription | 4 | 0.084689 | IL6, PDGFB, GATA2, TP53 |
| GO0009897~external side of plasma membrane | 12 | 0.085521 | SPN, CCR1, CXCR2, TLR8, SLAMF6, TRDC, LY6G5B, CXCR6, IL6R, TLR4, CSF2RA, CCR3 |
| hsa04657IL-17 signaling pathway | 5 | 0.085895 | NFKBIA, IL6, JUND, TRAF4, LCN2 |
| GO0035025~positive regulation of Rho protein signal transduction | 3 | 0.086141 | PRAG1, LPAR1, F2RL1 |
| GO0005788~endoplasmic reticulum lumen | 10 | 0.087127 | TOR4A, IL6, COLGALT2, IL23A, PDGFD, COL7A1, SDC2, PDGFB, CP, FSTL1 |
| GO0035580~specific granule lumen | 4 | 0.087553 | LCN2, PTX3, BPI, LTF |
| GO0042803~protein homodimerization activity | 19 | 0.088542 | ZNF397, EPM2A, ZNF396, CSF1R, HVCN1, RPE, IDH1, NOG, PDGFB, CISD1, DARS2, HSD17B4, NLRC4, ADRB2, KNSTRN, MSH2, HSD17B1, PNPO, IL6R |
| GO0001965~G-protein alpha-subunit binding | 3 | 0.090733 | GPSM2, LPAR1, F2RL1 |
| hsa04210Apoptosis | 6 | 0.091266 | NFKBIA, LMNA, TNFSF10, PRF1, TP53, TNFRSF1A |
| GO0034142~toll-like receptor 4 signaling pathway | 3 | 0.091298 | NFKBIA, SCIMP, TLR4 |
| GO0000922~spindle pole | 6 | 0.092973 | PPP2CB, CEP19, NEK7, SBDS, RMDN2, KNSTRN |
| hsa04213Longevity regulating pathway - multiple species | 4 | 0.095231 | HSPA1L, HSPA6, HSPA1B, HSPA1A |
| GO0045672~positive regulation of osteoclast differentiation | 3 | 0.096542 | CCR1, KLF10, IL23A |
| GO0051673~disruption of plasma membrane integrity in another organism | 2 | 0.097456 | DEFA1, DEFA1B |
| GO0051127~positive regulation of actin nucleation | 2 | 0.097456 | TRIM27, WHAMM |
| GO0010573~vascular endothelial growth factor production | 2 | 0.097456 | IL6, IL6R |
| GO0006569~L-tryptophan catabolic process | 2 | 0.097456 | KMO, IDO1 |
| GO0034354~'de novo' NAD biosynthetic process from L-tryptophan | 2 | 0.097456 | KMO, IDO1 |
| GO0007596~blood coagulation | 5 | 0.097856 | PRRG4, MMRN1, PROS1, FGL2, F2RL1 |
