# Supplementary material for: Soy protein alleviates DKD by restraining inflammation via the MAPKs/PPAR-γ signaling pathway
Source: Ren Fail. 2026 Jul 24;48(1):2698775. doi: 10.1080/0886022X.2026.2698775 (PMC13403453; doi:10.1080/0886022X.2026.2698775)
Supplement: Supplemental Material [file IRNF_A_2698775_SM2501.docx]

**Supplementary table 2.** Functional and pathway enrichment analyses of differentially expressed genes in the GSE139317 dataset.

| Term | Count | % | PValue | Genes |
| --- | --- | --- | --- | --- |
| GO:0006631~fatty acid metabolic process | 7 | 18.42105 | 6.10E-09 | CYP4A12A, CYP4A12B, CYP4A31, CYP4A10, ACOT1, LPL, ACOT3 |
| mmu01100: Metabolic pathways | 18 | 47.36842 | 6.36E-08 | CYP4A12A, CYP4A12B, MOGAT2, CYP4A31, CYP4A10, INMT, MTMR7, ADH1, BHMT, CYP2B10, KYNU, GSTA2, ACOT1, HMGCS2, GUSB, ALDH1A7, B4GALT5, ACOT3 |
| GO:0048252~lauric acid metabolic process | 4 | 10.52632 | 9.11E-08 | CYP4A12A, CYP4A12B, CYP4A31, CYP4A10 |
| mmu03320: PPAR signaling pathway | 7 | 18.42105 | 1.09E-07 | CYP4A12A, CYP4A12B, CYP4A31, CYP4A10, LPL, HMGCS2, ANGPTL4 |
| GO:0046456~icosanoid biosynthetic process | 4 | 10.52632 | 1.95E-07 | CYP4A12A, CYP4A12B, CYP4A31, CYP4A10 |
| GO:0008392~arachidonate epoxygenase activity | 5 | 13.15789 | 2.41E-07 | CYP4A12A, CYP4A12B, CYP4A31, CYP2B10, CYP4A10 |
| mmu00830:  Retinol metabolism | 7 | 18.42105 | 2.46E-07 | CYP4A12A, ADH1, CYP4A12B, CYP4A31, CYP2B10, CYP4A10, ALDH1A7 |
| GO:0052869~arachidonate omega-hydroxylase activity | 4 | 10.52632 | 2.90E-07 | CYP4A12A, CYP4A12B, CYP4A31, CYP4A10 |
| GO:0018685~alkane 1-monooxygenase activity | 4 | 10.52632 | 2.90E-07 | CYP4A12A, CYP4A12B, CYP4A31, CYP4A10 |
| GO:0050051~leukotriene-B4 20-monooxygenase activity | 4 | 10.52632 | 5.02E-07 | CYP4A12A, CYP4A12B, CYP4A31, CYP4A10 |
| GO:0102033~long-chain fatty acid omega-hydroxylase activity | 4 | 10.52632 | 9.81E-07 | CYP4A12A, CYP4A12B, CYP4A31, CYP4A10 |
| GO:0008391~arachidonate monooxygenase activity | 4 | 10.52632 | 2.32E-06 | CYP4A12A, CYP4A12B, CYP4A31, CYP4A10 |
| GO:0004497~monooxygenase activity | 5 | 13.15789 | 7.28E-06 | CYP4A12A, CYP4A12B, CYP4A31, CYP2B10, CYP4A10 |
| GO:0001676~long-chain fatty acid metabolic process | 4 | 10.52632 | 1.04E-05 | CYP4A31, CYP4A10, ACOT1, ACOT3 |
| mmu00071:  Fatty acid degradation | 5 | 13.15789 | 1.06E-05 | CYP4A12A, ADH1, CYP4A12B, CYP4A31, CYP4A10 |
| GO:0043651~linoleic acid metabolic process | 4 | 10.52632 | 1.34E-05 | CYP4A12A, CYP4A12B, CYP4A31, CYP4A10 |
| GO:0043231~intracellular membrane-bounded organelle | 9 | 23.68421 | 2.04E-05 | CYP4A12A, CYP4A12B, LIPO1, CYP4A31, CYP2B10, CYP4A10, MPV17L, GUSB, PPIC |
| GO:0019369~arachidonate metabolic process | 4 | 10.52632 | 2.90E-05 | CYP4A12A, CYP4A12B, CYP4A31, CYP4A10 |
| GO:0005615~extracellular space | 11 | 28.94737 | 4.01E-05 | GREM1, C1QTNF3, CYP4A12A, CYP4A12B, ANGPTL7, APOH, IL34, LPL, ANGPTL4, GUSB, JCHAIN |
| GO:0001822~kidney development | 5 | 13.15789 | 7.18E-05 | CYP4A12A, CYP4A12B, CYP4A31, CYP4A10, HMGCS2 |
| mmu00590:  Arachidonic acid metabolism | 5 | 13.15789 | 8.95E-05 | CYP4A12A, CYP4A12B, CYP4A31, CYP2B10, CYP4A10 |
| GO:0020037~heme binding | 5 | 13.15789 | 9.39E-05 | CYP4A12A, CYP4A12B, CYP4A31, CYP2B10, CYP4A10 |
| GO:0005506~iron ion binding | 5 | 13.15789 | 1.02E-04 | CYP4A12A, CYP4A12B, CYP4A31, CYP2B10, CYP4A10 |
| GO:0006629~lipid metabolic process | 5 | 13.15789 | 1.75E-04 | ADH1, MOGAT2, LPL, ANGPTL4, MTMR7 |
| GO:0016324~apical plasma membrane | 6 | 15.78947 | 2.84E-04 | CYP4A12A, CYP4A12B, CYP4A31, CYP4A10, ANXA13, SLC22A7 |
| GO:0035634~response to stilbenoid | 3 | 7.894737 | 3.25E-04 | SLCO1A1, GSTA2, SLC22A7 |
| GO:0019373~epoxygenase P450 pathway | 3 | 7.894737 | 4.55E-04 | CYP4A31, CYP2B10, CYP4A10 |
| GO:0033574~response to testosterone | 3 | 7.894737 | 0.001192 | ADH1, SLCO1A1, HMGCS2 |
| GO:0005789~endoplasmic reticulum membrane | 7 | 18.42105 | 0.001995 | CYP4A12A, CYP4A12B, CYP4A31, MOGAT2, CYP2B10, CYP4A10, MFSD2A |
| GO:0052689~carboxylic ester hydrolase activity | 3 | 7.894737 | 0.002383 | ACOT1, LPL, ACOT3 |
| GO:0120250~fatty acid omega-hydroxylase activity | 2 | 5.263158 | 0.002488 | CYP4A12A, CYP4A12B |
| GO:0016712~oxidoreductase activity, acting on paired donors, with incorporation or reduction of molecular oxygen, reduced flavin or flavoprotein as one donor, and incorporation of one atom of oxygen | 3 | 7.894737 | 0.002982 | CYP4A12A, CYP4A12B, CYP2B10 |
| GO:0042802~identical protein binding | 9 | 23.68421 | 0.003611 | C1QTNF3, ADH1, BHMT, ANGPTL7, APOH, IL34, HMGCS2, ANGPTL4, ALDH1A7 |
| GO:0032305~positive regulation of icosanoid secretion | 2 | 5.263158 | 0.003631 | CYP4A31, CYP4A10 |
| mmu04750: Inflammatory mediator regulation of TRP channels | 4 | 10.52632 | 0.005075 | CYP4A12A, CYP4A12B, CYP4A31, CYP4A10 |
| GO:0103002~16-hydroxypalmitate dehydrogenase activity | 2 | 5.263158 | 0.006208 | CYP4A12A, CYP4A12B |
| GO:0140981~medium-chain fatty acid omega-hydroxylase activity | 2 | 5.263158 | 0.006208 | CYP4A12A, CYP4A12B |
| mmu04270:  Vascular smooth muscle contraction | 4 | 10.52632 | 0.006742 | CYP4A12A, CYP4A12B, CYP4A31, CYP4A10 |
| mmu04979:  Cholesterol metabolism | 3 | 7.894737 | 0.008034 | APOH, LPL, ANGPTL4 |
| GO:0097267~omega-hydroxylase P450 pathway | 2 | 5.263158 | 0.009653 | CYP4A12A, CYP4A12B |
| GO:0005777~peroxisome | 3 | 7.894737 | 0.011993 | MPV17L, NUDT19, ACOT3 |
| GO:0016790~thiolester hydrolase activity | 2 | 5.263158 | 0.013609 | ACOT1, ACOT3 |
| GO:0042627~chylomicron | 2 | 5.263158 | 0.015635 | APOH, LPL |
| GO:0006691~leukotriene metabolic process | 2 | 5.263158 | 0.01564 | CYP4A31, CYP4A10 |
| GO:0005576~extracellular region | 7 | 18.42105 | 0.018971 | GREM1, C1QTNF3, IGH-VJ558, ANGPTL7, APOH, LPL, ANGPTL4 |
| GO:0042803~protein homodimerization activity | 5 | 13.15789 | 0.019454 | GREM1, KYNU, GSTA2, LPL, JCHAIN |
| GO:0005739~mitochondrion | 7 | 18.42105 | 0.020832 | ADH1, KYNU, GSTA2, MPV17L, NUDT19, ACOT1, HMGCS2 |
| GO:0006068~ethanol catabolic process | 2 | 5.263158 | 0.021592 | ADH1, ALDH1A7 |
| GO:0019432~triglyceride biosynthetic process | 2 | 5.263158 | 0.025147 | MOGAT2, LPL |
| GO:0047617~fatty acyl-CoA hydrolase activity | 2 | 5.263158 | 0.025824 | ACOT1, ACOT3 |
| GO:0055085~transmembrane transport | 3 | 7.894737 | 0.026992 | SLCO1A1, SLC22A7, MFSD2A |
| GO:0061098~positive regulation of protein tyrosine kinase activity | 2 | 5.263158 | 0.027509 | GREM1, IL34 |
| GO:0015347~sodium-independent organic anion transmembrane transporter activity | 2 | 5.263158 | 0.029459 | SLCO1A1, SLC22A7 |
| GO:0071398~cellular response to fatty acid | 2 | 5.263158 | 0.029866 | LPL, HMGCS2 |
| GO:0043395~heparan sulfate proteoglycan binding | 2 | 5.263158 | 0.031876 | GREM1, LPL |
| GO:0034361~very-low-density lipoprotein particle | 2 | 5.263158 | 0.032207 | APOH, LPL |
| GO:0006637~acyl-CoA metabolic process | 2 | 5.263158 | 0.035735 | ACOT1, ACOT3 |
| GO:0001523~retinoid metabolic process | 2 | 5.263158 | 0.035735 | ADH1, LPL |
| GO:0015711~organic anion transport | 2 | 5.263158 | 0.035735 | SLCO1A1, SLC22A7 |
| GO:0008514~organic anion transmembrane transporter activity | 2 | 5.263158 | 0.03909 | SLCO1A1, SLC22A7 |
| GO:0070328~triglyceride homeostasis | 2 | 5.263158 | 0.039239 | LPL, ANGPTL4 |
| GO:0009617~response to bacterium | 3 | 7.894737 | 0.0449 | CYP2B10, GSTA2, LPL |
| GO:0031100~animal organ regeneration | 2 | 5.263158 | 0.060007 | ADH1, APOH |
| GO:0005737~cytoplasm | 13 | 34.21053 | 0.069649 | CYP4A12A, CYP4A12B, CYP4A31, CYP4A10, MPV17L, ANXA13, MTMR7, ADH1, CYP2B10, KYNU, ACOT1, ALDH1A7, PPIC |
| GO:0006811~monoatomic ion transport | 2 | 5.263158 | 0.071355 | SLCO1A1, SLC22A7 |
| GO:0005829~cytosol | 10 | 26.31579 | 0.07163 | ADH1, BHMT, KYNU, GSTA2, ACOT1, ALDH1A7, INMT, MTMR7, ACOT3, MFSD2A |
| mmu00062: Fatty acid elongation | 2 | 5.263158 | 0.076008 | ACOT1, ACOT3 |
| GO:0016705~oxidoreductase activity, acting on paired donors, with incorporation or reduction of molecular oxygen | 2 | 5.263158 | 0.083583 | CYP4A31, CYP4A10 |
| GO:0015293~symporter activity | 2 | 5.263158 | 0.084726 | SLC6A15, MFSD2A |
| mmu01040: Biosynthesis of unsaturated fatty acids | 2 | 5.263158 | 0.088539 | ACOT1, ACOT3 |
| GO:0009925~basal plasma membrane | 2 | 5.263158 | 0.094725 | SLCO1A1, SLC22A7 |
| GO:0007596~blood coagulation | 2 | 5.263158 | 0.099147 | ANGPTL7, ANGPTL4 |
